# Conflict detection with invalid inferences: All heuristics, no logic

**DOI:** 10.3758/s13421-025-01709-w

**Published:** 2025-04-17

**Authors:** Veronika Kosourikhina, Simon J. Handley

**Affiliations:** School of Psychological Sciences, Macquarie University, Sydney, NSW 2109 Australia

**Keywords:** Conflict detection, Reasoning, Dual process theory, Conditional inference, Logical intuition

## Abstract

Under current process models of reasoning, detecting conflict between beliefs and logic is a key step that determines whether people will engage in reflective thinking. Conflict detection has been found across many tasks, but it is less reliably observed with syllogistic reasoning. ‘Reverse’ detection effects have also been found in some studies, which are not easily explained within the current model of conflict detection and cannot be attributed only to measurement noise. In this study, we test whether ‘reverse’ detection effects in invalid syllogisms can be attributed to a mismatch between where conflict is thought to occur based on normative expectations and where conflict actually occurs for participants. We present evidence from two experiments (total *N* = 248) showing that invalid AC and DA syllogisms are intuitively valid to many participants, and that there are generally no differences in reaction time and confidence between valid and invalid items once believability and chosen response are taken into consideration. Further, we show that ‘reverse’ detection effects on invalid items disappear if we treat them as valid, on the assumption that this reflects where conflict occurs subjectively. These results indicate that conflict in these items likely occurs between two heuristic responses, rather than logic and heuristics; and that subjective, not objective, conflict should be considered when measuring conflict detection.

**Supplementary information:**

The online version contains supplementary material available at 10.3758/s13421-025-01709-w.

## Introduction

Consider the following problem:

(1) *All flowers need water.*

*Roses need water.*

*Therefore, roses are flowers*.

This apparently simple logical inference is regularly endorsed (Evans et al., 2007, 2010), but a moment’s reflection might cause us to realise that, despite the conclusion aligning with our beliefs, the premise is not bi-directional. Such a realisation becomes obvious if we consider an unbelievable version of the same argument:

(2) *All flowers need water.*

*Humans need water.*

*Therefore, humans are flowers*.

Such simple logical inferences have been used to study how people resolve the conflict between beliefs and logic, a key issue addressed in dual-process accounts of reasoning. Dual process models view reasoning as an interplay between automatic, intuitive, Type 1 processes and controlled, reflective Type 2 processes. Earlier formulations of dual process theory proposed that responses based on beliefs or heuristics are evidence of Type 1, automatic processing, while logical responses are evidence for Type 2, reflective thinking. This simplification has been criticised on theoretical grounds (Evans & Stanovich, 2013; Keren & Schul, 2009) and has been challenged by empirical findings suggesting that logical responses can be fast and belief-based responses can be slow (Handley et al., 2011; Howarth et al., 2016; Newman et al., 2017; Trippas et al., 2017), and that people seem to detect conflict between belief-based and logical responses even when they cannot give the right answer (De Neys & Glumicic, 2008; Pennycook et al., 2012; Thompson et al., 2011). The theoretical critique and the empirical findings have led to the development of several parallel processing and hybrid models (De Neys, 2012; Handley & Trippas, 2015; Pennycook et al., 2015).

**Fig. 1:**
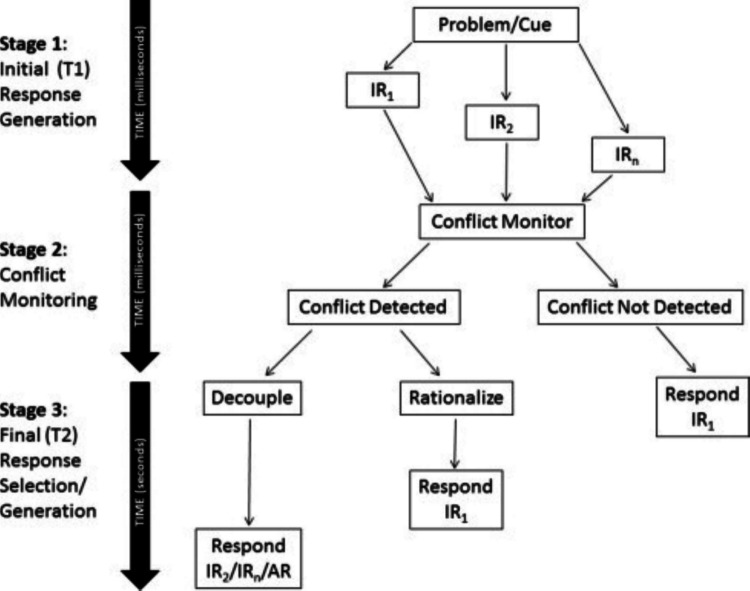
Figure from Pennycook et al. (2015). The diagram shows ‘intuitive’ processing (T1), conflict monitoring, and ‘analytic’ processing (T2). Intuitive responses (IRs) are produced with different response speeds, where IR_1_ is most salient is fluent. Alternative responses (ARa) can be generated if T2 is engaged

The most recent process model of reasoning is the three-stage model (Pennycook et al., 2015; Fig. 1). It integrates current findings in the literature and the critique of earlier dual-process models, to emphasise a key new element omitted by the previous models. In the three-stage model, it is proposed that a given problem can elicit multiple automatic responses with varying degrees of speed or activation strength. If one of the intuitive responses is stronger or faster than the other, it will be given as the answer without further deliberation. However, in cases where intuitive responses are more closely matched, reasoners could notice the conflict between the response options. Participants may detect the conflict but shortcut to giving the dominant response or detect the conflict and engage reflective controlled processes to find the correct response (Bago & De Neys, 2017; De Neys & Pennycook, 2019; Pennycook et al., 2015).

In this view, conflict detection is the key element that determines whether reflective processes are engaged at all. This separates it out as a distinct element from reflective processing itself. This was a major problem for earlier default-interventionist dual-process models, which could not explain how reasoners could switch between giving automatic responses and reflective thinking (the so-called ‘switch problem’; De Neys, 2023). Understanding the mechanism of conflict detection is an important precursor to understanding the conditions in which people are more likely to engage in effortful, reflective thinking that may lead them to conclusions that differ from their baseline beliefs or otherwise challenge their intuitions.

### Conflict detection and intuitive logic

A controversial claim in these dual-process accounts of reasoning is that intuitive processes not only lead to bias but are also sensitive to the logical status of an argument. The intuitive logic hypothesis claims that on certain simple, common reasoning tasks, people often generate the logical, normative response automatically, but this intuitive response comes into conflict with other intuitions, such as those based on conclusion believability, which often prevents participants from providing the logic-based response (De Neys, 2012, 2014). This claim draws upon evidence that reasoners take longer and are less confident on belief-logic conflict problems, irrespective of whether they give the correct logical response.

Comparing problems with and without belief-logic conflict shows that people are generally sensitive to conflict, even if they give a biased response and not a logical one. Differences between conflict and no-conflict problems have been demonstrated with behavioural (reaction time: De Neys & Glumicic, 2008; Pennycook et al., 2012), metacognitive (confidence ratings: Ackerman & Thompson, 2017; Johnson et al., 2016; Thompson et al., 2011), and physiological measures (EEG and fMRI: Banks & Hope, 2014; Goel & Dolan, 2003). Longer response times or a decrease in confidence on a conflict item relative to no-conflict items is thought to indicate that participants engaged in some deliberation, which suggests that conflict detection has occurred.

Further evidence has accumulated showing that participants seem to be able to give logical or normative responses automatically. The presence of logical intuitions has been demonstrated with the two-response paradigm, which shows a subset of participants are able to give normative responses under a tight time limit or a cognitive load which should prevent participants from reflecting on the problem (Bago & De Neys, 2017; Raoelison et al., 2020; Raoelison & De Neys, 2019; Thompson et al., 2011). This view has also been supported by research using the ‘liking paradigm’, where participants rate logically valid statements as more likeable or brighter than invalid statements, regardless of believability (Morsanyi & Handley, 2012; Nakamura & Kawaguchi, 2016; Trippas et al., 2016), and studies showing that judgements concerning the believability of a given conclusion are influenced by its validity (Handley et al., 2011; Newman et al., 2017; Trippas et al., 2017).

Based on this body of work researchers have argued (e.g., De Neys et al., 2011, 2013; De Neys & Glumicic, 2008; Stupple & Ball, 2008), that conflict detection is automatic and successful even in biased participants, and bias primarily arises from the failure to engage controlled Type 2 processing or inhibit belief-based responses (Bonner & Newell, 2010; De Neys, 2014; De Neys & Bonnefon, 2013). Work on intuitive logic has been critical to moving away from the default-interventionist perspective, which tended to ascribe biased responses to Type 1 processing and normative responses to Type 2 (an approach that has been criticised by the authors of the theory; Evans & Stanovich, 2013). The model of conflict detection that emerged from this work (De Neys & Pennycook, 2019) does not explicitly claim that a logical intuition must be present; it is typically assumed in practice that the conflict being studied is between intuitive beliefs and intuitive logic. In this sense, conflict detection is in practice defined as a mechanism that corrects biases arising from incorrect intuitive beliefs through the application of correct intuitive logic, although in theory it could be a mechanism for resolving conflict between any kind of inconsistent automatic responses.

The limitations of this narrow definition of conflict detection begin to show once we examine the idea of logical intuitions more closely, as well as in light of recent empirical findings showing that ‘intuitive logic’ effects may have nothing to do with logic but instead arise through the operation of heuristics. One major source of support for the idea of logical intuitions are studies in which participants are instructed to respond based upon conclusion features that are independent of logical validity. Studies utilising this approach have shown that judgements of conclusion believability (e.g., Handley et al., 2011; Trippas et al., 2017), likeability (Morsanyi & Handley, 2012; Trippas et al., 2016), or even brightness (Ghasemi et al., 2021; Trippas et al., 2016) are influenced by item validity. This suggests that the logical structure of the item is processed automatically and does not require deliberation, otherwise it should not impact these unrelated types of judgement.

However, recent work suggests that, at least in some cases, intuitive logic effects are better explained as arising from the operation of surface-level heuristics that happen to produce responses that align with logic in the studied materials. For example, on the conjunction task, conflict items tend to have more similar probabilities associated with each of the response options, as compared to no-conflict items, which has been argued to reduce confidence in the responses (Aczel et al., 2016). Logic liking effects disappear when the materials control for surface features, such as atmosphere (Ghasemi et al., 2023; Meyer-Grant et al., 2023), repetition (Klauer & Singmann, 2013), or argument strength (Ghasemi et al., 2022). This suggests that heuristics, rather than intuitive sensitivity to logical structure, influenced liking judgements in earlier studies.

The effect of validity on liking judgements has also been shown to be moderated by working memory capacity and application of cognitive load, such that participants with more limited working memory availability had more difficulty distinguishing between valid and invalid items, both when making judgements of validity and liking (Ghasemi et al., 2021; Hayes et al., 2020). This suggests that logic liking effects are sometimes influenced by Type 2 controlled processes which are thought to be defined by working memory engagement (Evans & Stanovich, 2013). This contradicts the claim that preferring valid items in liking judgements reflects an intuitive processing of their logical structure. Controlling for perceptual sensitivity also diminished the logic-brightness effects (Hayes et al., 2022), similarly undermining the claim that logic is intuitively processed to the degree that it influences other unrelated types of judgements.

The idea of logical intuitions is key to the current class of dual-process models (known as DPT 2.0, De Neys, 2018). While earlier dual-process models claimed that normative responses were necessarily the result of slow, reflective thinking, the discovery of logical intuitions allowed theorists to claim that reflective thinking was not necessary, and normative responses could be produced fast and without taxing cognitive resources. The studies described above cast some doubt on this claim, instead suggesting that participants produce intuitive responses aligned with normative standards through heuristics rather than genuine sensitivity to logical principles.

These findings provide a challenge to the interpretation of evidence from the conflict-detection paradigm, evidence that has been identified as providing key support to DPT 2.0. In the conflict-detection paradigm, items are constructed to create conflict between logical and belief-based components of the problem, and conflict detection is theorised to occur between the logical intuition and the belief-based response. If logical intuitions are not necessarily based on sensitivity to the logical features of the problem, then the designation of ‘conflict’ and ‘no-conflict’ items based upon assumptions about which logical and belief-based responses they elicit may no longer be reliable. In the following sections we present a case where we expect the ‘logical intuition’ to be misaligned with formal logic. In this case we would expect the opposite effect to what might be expected in a standard conflict detection study. We consider what this might mean for our understanding of how people notice errors in their reasoning.

### ‘Reverse’ detection in AC (Affirmation of the Consequent) and DA (Denial of the Antecedent) conditional syllogisms

Conditional syllogisms are a widely used type of reasoning task, one where logical intuitions are expected to be ubiquitous (De Neys, 2012, 2014). They can vary substantially in structure and content along many dimensions that have been shown to impact accuracy (e.g., Evans et al., 1983, 1999; Thompson, 1994). In a typical reasoning task participants are presented with valid arguments (Modus Ponens, MP; and Modus Tollens, MT) and invalid arguments (Affirmation of the Consequent, AC; or Denial of the Antecedent, DA) in which the believability of the conclusion is systematically manipulated. In a recent study, Ghasemi et al. (2022) utilised the belief/logic instructional paradigm and included arguments that are formally valid, such as MP (*if P then Q, P, therefore Q*), and invalid versions of the same arguments (*if P then Q, P, therefore not Q*). In line with previous studies, the research showed that argument validity impacted judgements concerning the believability of the conclusion, consistent with the claim that the logical inference was activated automatically, hence interfering with a conclusion belief judgement. Ghasemi et al., also included invalid conditionals, such as AC, which are commonly endorsed as valid (*if P then Q, Q, therefore P*), together with versions of the same argument that are less commonly endorsed (*if P then Q, Q, therefore not P*). These argument forms were labelled as pseudo-valid and pseudo-invalid, respectively. Importantly, the research showed an effect of pseudo-validity on belief judgements – reasoners found belief judgements to be more difficult when the believability of the conclusion conflicted with the ‘pseudo-validity’ of the argument.

Ghasemi et al. (2022, 2023) argued that if logical intuitions were based upon formal logical principles, then validity should only impact belief judgements on valid problems, but not on invalid AC and DA problems. They further argued that the observed ‘logical intuitions’ were underpinned by the operation of a matching heuristic. The matching heuristic involves participants pairing positive or negative elements that repeat in the premises and the conclusion (e.g., *if P then Q, Q, therefore P*; or, *if P then Q, not P, therefore not Q*) and concluding that arguments with such paired elements must be valid. This is in contrast to cases where the premises and conclusion use elements with different polarities, which participants tend to reject (e.g., *if P then Q, P, therefore not Q*). A similar argument has been made by Meyer-Grant et al. (2023), who claimed that the logic liking effects arose because of an atmosphere effect linked to a match between the conclusion and premises. Such matching heuristics have long been claimed to play a role in determining responses to reasoning tasks (Wason, 1968; Wetherick & Gilhooly, 1995).

AC and DA conditionals are also often endorsed as valid on arguments with abstract content (Evans et al., 2007, 2010) – less often than valid MP and MT inferences, but at a high rate nonetheless (~60–70% for AC and ~40–60% for DA in the above studies). The use of a matching heuristic would align with high endorsement rates on these items. Endorsement rates on AC and DA conditionals further vary depending on counterexample availability, which varies depending on how materials are designed and on individual differences. Participants are more likely to draw the normatively correct conclusion that these items are invalid if the materials allow them to come up with counter-examples more easily (Newman et al., 2017; Rumain et al., 1983; Thompson, 1994) or because they adopt a thinking strategy that prioritises the search for counterexamples (Markovits et al., 2017). If acceptance rates differ depending on materials and individual differences, then the endorsement of conclusions to invalid inferences is unlikely to arise from a well-automated logical intuition and a matching heuristic is one potential explanation of the responses.

An alternative view is that reasoners are logical in principle but err in practice because of the limitations of working memory. The Mental Model Theory (MMT) argues that reasoners understand conditionals by constructing mental models corresponding to the states of affairs described in the premises (Johnson-Laird & Byrne, 1991). However, due to processing limitations, models are often incomplete, at least initially. Consider, for example, the conditional, ‘if P then Q’, according to MMT reasoners construct an initial model corresponding to just the p and q elements:

[P]   Q:  ...

where the ellipsis ‘…’ indicates that there are other models consistent with the conditional that are not initially represented. The exhaustion symbol ‘[P]’ indicates that P is exhausted with respect to Q and hence any remaining possibilities are ones in which ‘not-P’ holds. If reasoners have sufficient time and/or working memory capacity, they may flesh out the remaining possibilities, making explicit that the conditional is consistent with the following three models:

P: Q
not-P: Q
not-P: not-Q

However, reasoners will often fail to flesh out the full set of possibilities and reason based on only the initial model. Hence given the AC categorical premise ‘Q’, they will tend to infer that ‘P’ follows, because they have failed to flesh out the second model, which provides a counterexample to this conclusion. So according to MMT, the endorsement of the conditional fallacies arises from a reasoning process, but one that is incomplete, as it relies on the construction of an initial and superficial representation of the premises.

The MMT has been criticised on empirical and theoretical grounds (Evans et al., 2005; Evans & Over, 2004; Over et al., 2007). More recently, a number of alternative accounts of conditionals have been developed which draw upon probabilistic principles, rather than logic, as a normative standard. Such approaches include the suppositional account of conditionals (Evans et al., 2005), which proposes that reasoners understand conditionals through engaging in a mental simulation, in which they temporarily suppose the antecedent condition holds and evaluate their degree of belief in the consequent in light of this supposition (Evans et al., 2003). According to this account, conditional inferences will depend in large part on their relevant conditional probabilities, for example, P(q/p) for MP and the P(p/q) for AC. Hence, invalid inferences such as AC might often be drawn because they are probabilistically strong (P(p/q) is high), rather than logically entailed. We will return to such probabilistic accounts in the general discussion of this paper.

Irrespective of whether invalid conditional inferences are endorsed because of a matching heuristic, the failure to flesh out an initial model or because they are judged to be probabilistically strong, the observation that these inferences are regularly drawn has meaningful consequences for research on conflict detection. Such research is based on a model which assumes that reasoners have access to logic-based intuitions which often conflict with belief-based responses (De Neys & Pennycook, 2019), presumably based on formal rules of inference commonly associated with the direct and indirect mental proofs common in natural deduction accounts of human reasoning (Braine & O’Brien, 1998; Rips 1994). The evidence that AC and DA arguments are often endorsed as valid by participants would influence the analysis of and understanding of conflict detection effects in a way that has not currently been considered in the literature. For example, consider again argument (1) presented at the beginning of this paper, an invalid AC argument with a believable conclusion. Such items are typically considered to be conflict problems, but if the generated intuition is that the conclusion follows, then there will be no conflict between the ‘logical intuition’ and the believability of the conclusion – the conflict item would not elicit conflict detection. In contrast, consider argument (2), an invalid AC argument with an unbelievable conclusion. In this case the ‘logical intuition’ that the conclusion follows would conflict with the belief status of the conclusion and elicit conflict detection.

If the conflict and no-conflict items shown in Table 1 are in fact are mis-characterised in this way, this would create a ‘reverse’ conflict detection effect, arising from the mismatch between where an experimenter expects conflict to happen and where it actually happens. ‘Reverse’ detection effects happen when participants are faster or more confident on conflict than no-conflict items (i.e., the opposite to the usual pattern). They occur in a range of tasks (e.g., base-rate task: Pennycook et al., 2015; ratio bias task: Mevel et al., 2015; Cognitive Reflection Test: Travers et al., 2016; syllogisms: Teovanović, 2019), and cannot be explained away as mere noise (Pennycook et al., 2015). In case of syllogisms in particular, reverse detection was the predominant effect on invalid AC and DA conditionals, while valid MP and MT syllogisms generally show a true detection effect (Brisson et al., 2018).

**Table 1:** Examples of conflict and no-conflict AC (Affirmation of the Consequent) and DA (Denial of the Antecedent) items

		AC	DA
Conflict	Invalid-believable	All plants need water Roses need water Roses are plants	All fruits can be eaten Knives are not fruits Knives cannot be eaten
No-conflict	Invalid-unbelievable	All whales are mammals Mammals can walk Whales can walk	All birds can fly Planes are not birds Planes cannot fly

### Nature of conflict: Objective versus subjective

Earlier theorising defined conflict detection as occurring between an intuitive response and a response arising from normative rules (e.g., De Neys, 2012; Stanovich et al., 2016, p.43). In this view, what is being detected is one’s own bias. Similar to formulations of the default-interventionist dual-process models of the time, there was an implicit assumption that normative responses are somehow available to participants, but are overshadowed by the strong heuristic response elicited by the task. In the case of dual-process models, this was reflected in a common misconception that normative responses are evidence of Type 2 processing – which has been challenged by a number of theorists (De Neys, 2023; Evans & Stanovich, 2013). The concept of conflict detection and research demonstrating ‘intuitive logic’ effects has been interpreted as showing that normative responses can be given through Type 1, automatic processing.

Conflict detection has been reformulated to refer to conflict between assorted intuitive responses more generally (e.g., Boissin et al., 2023; Voudouri et al., 2022), such that what is being detected is inconsistency or incongruence between intuitive responses. Theory has been modified to allow any kind of intuitions to be involved in conflict detection, not necessarily involving intuitive logic (De Neys, 2023; Pennycook et al., 2015). However, the method for estimating conflict detection remains the same, and hinges on assumptions that may be incorrect if the dominant ‘logical intuition’ is misaligned with normative principles.

This has implications for the measurement of conflict detection and understanding how it works. Theorists pointed out early on that it is not assumed that everyone always has a logical intuition available (De Neys, 2014), but that it is common enough to safely assume that conflict between logical and belief-based responses generally occurs as expected. Conflict detection measurement relies on the assumption that objective and subjective conflict generally match – because the results of many studies have generally found the difference in the expected direction, in most tasks. For the purposes of simplifying experiments, this is a sound tactic, because it allows researchers to rely on simple, fixed points of comparison. However, if conflict does not occur where experimenters expect it to, the results can become difficult to interpret or misleading.

### The present study

The evidence suggests that AC and DA conditional arguments are regularly endorsed leading people to perceive these arguments as ‘valid’ unless there are counterexamples available. If this is the case, and the corresponding logical intuition does not align with formal logic, then objective and subjective conflict on AC and DA conditionals will be dissociated.

In this paper, we set out to demonstrate the presence of ‘reverse’ detection effects on AC and DA syllogisms. ‘Reverse’ detection effects in syllogisms have been reported in one previous study that we know of (Brisson et al., 2018), which included two experiments investigating the effects of syllogism complexity on conflict detection. Conflict detection was measured only with reaction time (RT), where items were presented either in full, or in parts to allow for more precise response time measurement. It is difficult to say how common reverse detection effects on AC and DA items are because studies either do not use the same types of syllogisms (e.g., Mata et al., 2013; Prowse Turner & Thompson, 2009; Trippas et al., 2017) or do not report detection effects broken down by syllogism type (e.g., Bago & De Neys, 2017; Thompson et al., 2011). To confirm that these are reliable effects, we tested for the presence of ‘reverse’ detection effects using both conditional (Experiment 1) and categorical syllogisms (Experiment 2).

Previous studies have utilised RT, confidence judgements, or both to measure conflict detection. When used concurrently, the two measures were found to sometimes disagree (Burič & Šrol, 2020; Frey et al., 2018; Šrol & De Neys, 2020). Reaction time can be considered more objective, as it does not involve self-report. However, confidence is a more direct measure of the metacognitive uncertainty thought to drive conflict detection, while response fluency reflected in RTs is only one component of it (Ackerman, 2019; Koriat, 2012). We report both measures for completeness, although we believe confidence judgements to be a more theoretically grounded measure.

Second, we investigate how AC and DA arguments are responded to intuitively. We use a speeded abstract task to gauge the intuitive responses associated with these items, and consider how this relates to responses on the main task.

We also investigate how confidence and RT vary depending on whether participants endorse the conclusion as valid. Our key hypotheses are detailed below:

- H1: We predict reverse detection effects on AC and DA items, such that invalid no-conflict items will be solved with higher accuracy, but also show longer response times and lower confidence than conflict items.
- H2: We predict that participants who endorse more of the abstract AC and DA items as valid will also endorse more of the AC and DA items as valid on the main task.
- H3: We expect participants to show similar RTs and confidence on ‘yes’ and ‘no’ responses to valid and invalid items, given that there will be an intuitive tendency to treat all items as valid.

#### Transparency and openness

All data exclusions, manipulations, and measures are reported following JARS. All study materials, data, and analysis scripts are available on the Open Science Framework (OSF) (osf.io/msdn2). Package versions for all R packages are listed in the analysis file. The design and analysis were not preregistered. Sample size was determined based on recommendations from Brysbaert (2019).

## Experiment 1

In this experiment, we investigate the automatic responses to AC and DA items to determine whether participants respond as if they are intuitively. We check whether AC and DA conditional syllogisms produce ‘reverse’ detection effects through RT and confidence, and whether these can be accounted for by considering how these items are perceived by participants.

### Method

#### Participants

Participants for all experiments were recruited from Macquarie University first-year psychology pool. Participants received course credit, and the study received ethics approval. All experiments were hosted on Pavlovia. For this experiment, we recruited 97 participants (80% identified as female, mean age = 21.2 years). Ninety-four participants were left after exclusion criteria were applied. In addition to syllogisms and the speeded abstract reasoning task, participants completed a stop-signal task and another speeded task evaluating the subjective believability of the conclusions as part of another study not presented here.

#### Materials and procedure

##### Conditional syllogisms

We used four types of conditional syllogisms: MP, MT, AC, and DA. Example items are shown in Table 2. MP and MT items are valid, and are typically easier for participants to generate the logical solution compared to AC and DA which are invalid inferences but nevertheless regularly endorsed (Brisson et al., 2018; Evans et al., 2007, 2010). To create unbelievable conclusions, we started from untrue premises (e.g., “*If an animal meows, then it’s* *a dog.*”). This allowed us to keep negation implicit in MT and DA items, to avoid biases that can be introduced from explicit negation (Evans & Handley, 1999; Schaeken & Schroyens, 2000).

**Table 2:** Example syllogisms used in Experiment 1

Conflict			No-conflict	
MP	VU	If a cat is angry, then it will purr. The cat is angry. Therefore, it will purr.	VB	If a bird is black, then it’s a raven. The bird is black. Therefore, it’s a raven.
MT	VU	If an animal has hooves, then it is a rabbit. It is a giraffe. Therefore, it has paws.	VB	If an animal hates swimming, then it’s a cat. The animal is a fish. Therefore, it loves swimming.
AC	IB	If an animal has a beak, then it’s a bird. It is a bird. Therefore, it has a beak.	IU	If an animal meows, then it’s a dog. The animal is a dog. Therefore, it meows.
DA	IB	If an animal is a bird, then it lays eggs. It is a mammal. Therefore, it gives live birth.	IU	If an animal is a whale, then it lives in water. The animal is a fish. Therefore, it lives on land.

V/I = valid, invalid; B/U = believable, unbelievable

We used eight items of each type and conflict level (8 x 2 conflict levels x 4 types = 64 items) and eight practice items, making it a total of 72 items in the syllogistic reasoning task. Participants were instructed to assume that the premises are true, and only assess whether the conclusions necessarily follow from the premises. The instructions included an invalid believable categorical syllogism, but did not specify what the correct answer is.

We used the step-by-step presentation format introduced by Pennycook et al. (2015) to minimise the influence of reading speed on measured response RT. The first and second premise were presented together for 3,000 ms, followed by whole syllogism presentation with free response time, which was recorded for analyses. A fixation cross was presented for 500 ms between trials. There were also two breaks during each syllogism task (after 20th and 40th trials), to encourage better focus.

##### Confidence

Participants were asked how confident they felt about their response after every trial, and presented with a slider ranging from 0 (not at all confident) to 100 (completely confident). We refer to conflict detection index based on confidence as CD-FOR, in line with previous work (short for ‘feeling of rightness’).

##### Speeded abstract reasoning task

The task included 20 conditional syllogisms, with five items from each of the four syllogism types (MP, MT, AC, DA). See Table 3 for example items. There was one real category (plants, fish, mammals, etc.), a second non-word category, and a non-word instance of one of the categories. Non-words were three- to five-letter words with only legal bigrams (Morsanyi & Handley, 2012; Rastle et al., 2002, www.cogsci.mq.edu.au/research/resources/nwdb/nwdb.html).

**Table 3:** Example items from the speeded abstract reasoning task

Valid		Invalid	
MP	If an animal is a von, then it is plart It is a von Therefore, it is a plart	AC	If a reptile is a yesp, then it is a saig It is a saig Therefore, it is a yesp
MT	If a bird is a mets, then it is glec It is not a glec Therefore, it is not a mets	DA	If a fish is a feeke, then it is a zorm It is not a feeke Therefore, it is not a zorm

Each item was presented for 7 s and participants saw a timeout bar above each item showing a decreasing block of colour to indicate remaining time. If the participant did not respond within the time limit, their response was recorded as incorrect. The time limit was calculated based on average RTs in a similar task conducted for a different study not presented here (mean + 1 SD), where participants were also instructed to give quick intuitive responses, and similarly to previous studies that imposed time limits on syllogisms to elicit intuitive responding (Voudouri et al., 2022; 4,000-ms reading time, 3,000-ms response time). Mean RT on the speeded abstract task here was 3.8 s (range 0–7 s), as compared to 5.6 mean RT on the main task (range 0–361 s).

##### Exclusion criteria

We excluded syllogism task data if a participant had < 75% accuracy on no-conflict MP problems, which removed data for three participants. Reaction times were positively skewed, so we log-transformed them, as is the usual recommendation (Baayen & Milin, 2010). We removed 14 log-RT data points trials that were unusually fast (< 3 SD from mean RT for a given participant), to filter out accidental or purposeful trial skips.

##### Conflict-detection indexes

We calculated a baseline value for each participant and syllogism type from correct no-conflict items. We excluded items that had RT > 50,000 ms or confidence < 25%, because we considered these unrepresentative of truly no-conflict cases. Fifty seconds is well above the mean RT (5.6 s), and low confidence on no-conflict items is either accidental or suggests participants had doubts about their answer and so could have experienced conflict. This removed 20 trials from RT data, and 50 trials from confidence data (0.9% and 2.4% of this subset). The appropriate baseline was then subtracted from log-RTs and confidence estimates on conflict items. We calculated conflict detection for all incorrect conflict trials, separately for each syllogism type for each participant. It is expected that participants will be slower and less confident on conflict items, so positive CD-logRT values and negative CD-FOR values indicate conflict detection.

### Results

We used R for all analyses, version 4.1.3. Key analyses were done with mixed effects models. The predictor variables are called fixed effects and any factors that are only included to account for noise (e.g., participant IDs, item number or any other grouping variables) are called random effects (Brauer & Curtin, 2018). We used the likelihood ratio test to evaluate effect significance.

In key analyses, we apply the Bayesian parameter estimation approach (Kruschke & Liddell, 2018) in addition to the mixed effect models. The results replicate our main findings, so we present these analyses in the supplementary materials for brevity. The code for all analyses is available on the OSF page for this project (https://osf.io/msdn2/).

#### Accuracy

Modelling the effects of conflict and validity on accuracy, we found significant main effects of conflict (*χ*^*2*^(1) = 28.05, *p* < .001) and validity (*χ*^*2*^(1) = 34.17, *p* < .001), but no interaction effect (*p* = .14). Participants were more accurate on no-conflict than conflict items (0.77 vs. 0.42), and more accurate on valid than invalid items (0.79 vs. 0.39). Overall, accuracy was clearly influenced by the presence of belief-logic conflict.

#### Reaction time

**Fig. 2:**
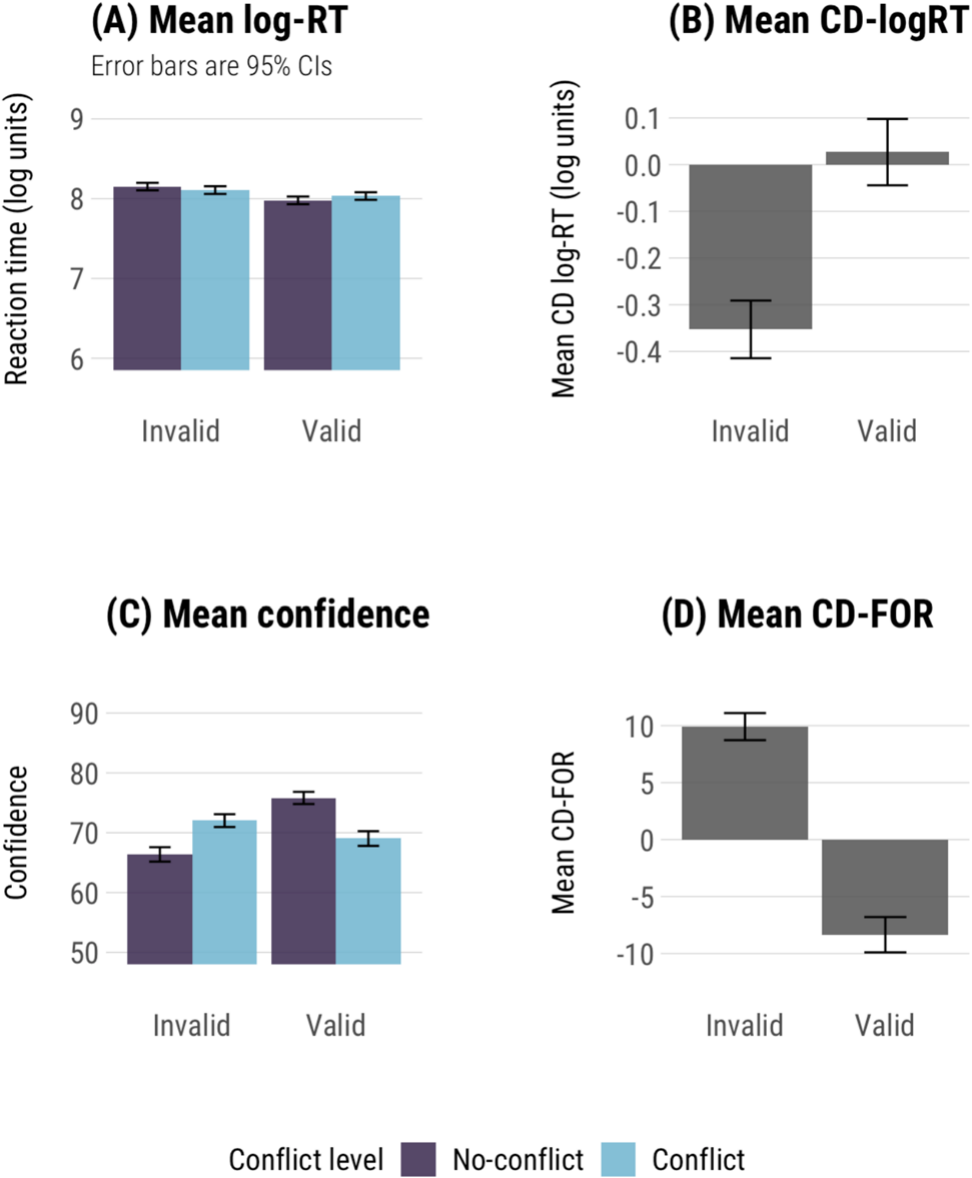
Mean log-RT, conflict detection index with log-transformed reaction times (CD-logRT), confidence, and conflict detection index based on confidence judgements (CD-FOR). Error bars are 95% confidence intervals

Modelling the effect of conflict and validity on log-transformed RT showed very small differences between conflict and no-conflict items (Table 4, Fig. 2a). There were no significant effects of conflict (*p* = .97), validity (*p* = .10), or their interaction (*p* = .52) on RT. There were no differences in mean log-RTs between conflict and no-conflict items (8.07 vs. 8.07) and a trend towards longer RTs on invalid than valid items (8.13 vs. 8.01). In Fig. 2b, we can see that valid items show no conflict detection effect based on log-RT, but invalid items show a reverse detection effect. To verify whether CD-logRT effects are different from zero on invalid but not valid items as we saw in Fig. 2b, we used single t-tests for valid and invalid item data separately. CD-logRT was significantly smaller than zero on invalid items (*t*(989) = −10.81, *p* < .001), but not on valid items (*p* = .47).

**Table 4:** Mean accuracy, log-RT, and confidence judgements

		Accuracy (%)	Log-RT (SE)	Confidence (SE)
Valid	No-conflict	82	7.98 (0.02)	76 (0.52)
	Conflict	56	8.03 (0.02)	69 (0.63)
Invalid	No-conflict	51	8.15 (0.02)	66 (0.61)
	Conflict	28	8.11 (0.02)	72 (0.55)

#### Confidence

We again modelled the impact of conflict and validity, now looking at confidence. The differences between conflict and no-conflict items were more pronounced in terms of confidence ratings (Fig. 2c). There was no significant effect of conflict on confidence (*p* = .58), but there was a main effect of validity (*χ*^*2*^(1) = 8.82, *p* = .003) and an interaction effect (*χ*^*2*^(1) = 27.82, *p* < .001). Conflict and no-conflict items had very similar confidence overall (70.5 vs. 71.1), while the difference between valid and invalid items was larger (72.4 vs. 69.2). Pairwise contrasts showed that valid no-conflict syllogisms were solved with more confidence than conflict valid syllogisms (*b* = 6.79, *p* < .001), but invalid no-conflict syllogisms were solved with less confidence than conflict items (*b* = −5.62, *p* < .001). To confirm that CD-FOR effects on valid and invalid items both differ from zero as we saw in Fig. 2d, we used single-sample t-tests. Mean CD-FOR values showed an overall conflict detection effect in the expected direction for valid syllogisms (*t*(620) = −10.435, *p* < .001), and a reverse detection effect for invalid items (*t*(979) = 15.678, *p* < .001).

#### Summary

While there were clear differences in accuracy between conflict and no-conflict items, there was no overall difference in RT or confidence. Pairwise contrasts showed that valid items had higher confidence on no-conflict items, as expected, but invalid items showed a reversal, with lower confidence on no-conflict items than conflict items. This reversal was also seen in conflict detection effects on invalid items, both with confidence and RT.

#### What is the intuitive response to AC and DA conditional syllogisms?

To investigate the predominant response to invalid syllogisms, we looked at how participants responded on a speeded abstract task, and whether they respond the same way to valid and invalid items in the main task. When analysing responses, “yes” answers indicate that the participant thought the conclusion was valid, and “no” answers indicate the conclusion was invalid.

First, we examined how participants responded to abstract items and how their performance on these items relates to performance on standard syllogisms. Abstract items were designed to be belief-neutral, and were completed under time pressure (7 s per item), so we consider responses on these items are an indicator of participants’ intuitive judgements, and suggest how a participant would likely respond to a given item type in the absence of the believability manipulation applied to the main item set. The task only records how consistently and automatically people respond to a given type of problem.

On average, valid and invalid abstract items were endorsed at a similar rate: 73% of responses on valid items endorse the conclusion as valid, and 66% of responses on invalid items also endorse the conclusion as valid. Modelling ‘yes’ responses by validity, there were no significant differences between responses to valid and invalid items (*p* = .06), meaning that participants generally did not differentiate well between abstract forms of valid and invalid syllogisms under time pressure. This can be interpreted as an absence of an intuition aligned with formal logic for these invalid items, or, in other words, that invalid items are subjectively valid for most participants in the absence of belief-based components pushing them one way or another.

**Fig. 3:**
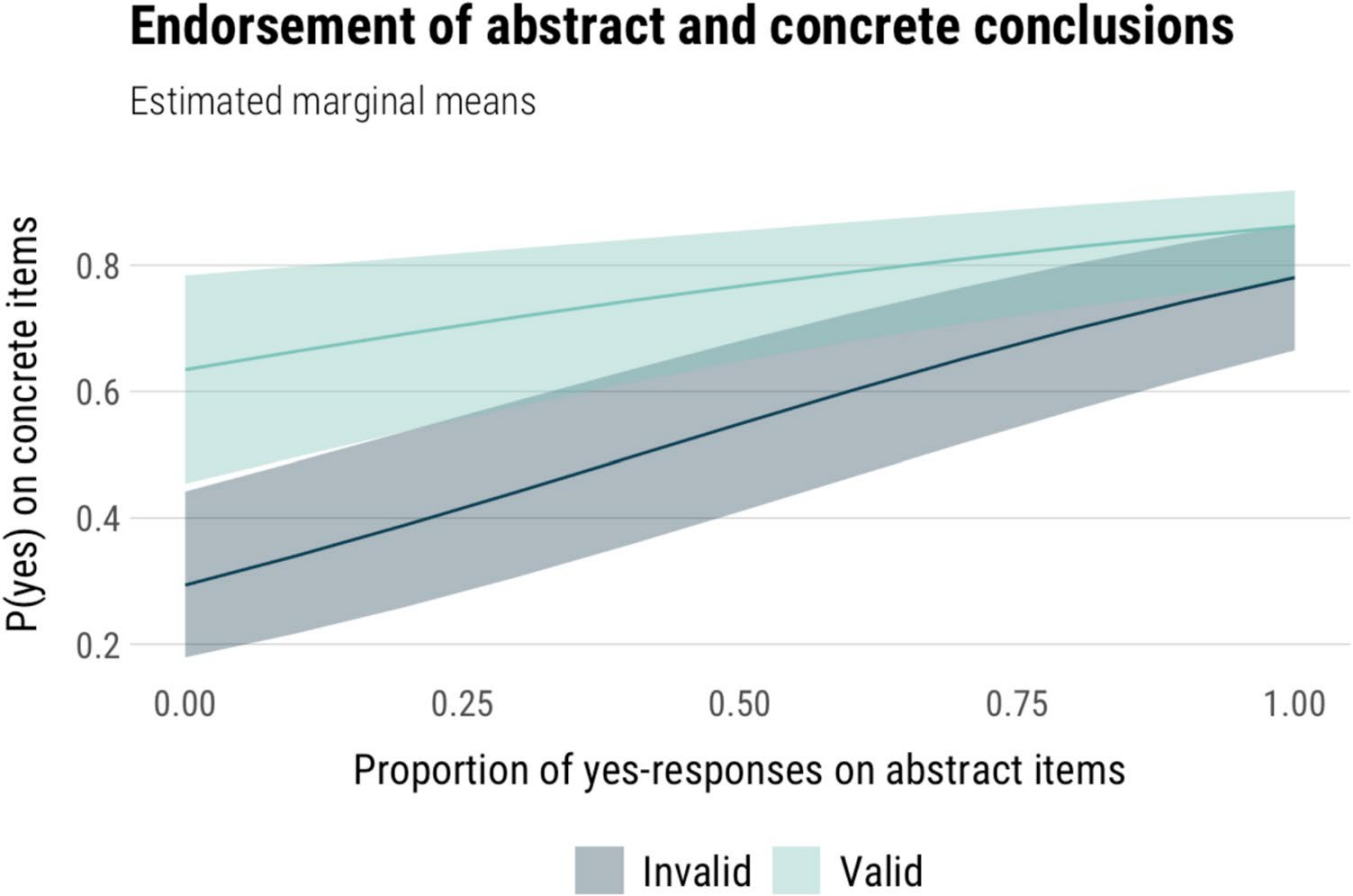
Estimated marginal means of the model predicting yes-responses on syllogisms from yes-responses on an abstract syllogism task and item validity

*Does logical intuition predict responses?* Next, we modelled the relationship between the proportion of conclusions each participant endorsed as valid on the main syllogism task and the abstract task, looking at both valid and invalid items. Response (yes, no) on the main task was the dependent variable, and validity and proportion of yes-responses on the abstract task were the independent variables. Proportion of yes-responses was computed separately for valid and invalid abstract items. The estimated model effects are shown in Fig. 3.

Responses on the main task were predicted both by the proportion of conclusion endorsements on the abstract task (χ^2^(1) = 43.58, *p* < .001), item validity (*χ*^*2*^(1) = 10.19, *p* = .001), and their interaction (*χ*^*2*^(1) = 7.31, *p* = .007). We used pairwise contrasts to more closely look at the relationship between main and abstract task conclusion endorsement for valid and invalid items separately. Participants were more likely to endorse the conclusion of the main task item as valid when they have endorsed more of the abstract item conclusions as valid, on both valid (*OR* = 3.60, *p* < .001) and invalid items (*OR* = 8.55, *p* < .001) of the main task.

#### Relationship between RT, confidence, and the chosen response

**Fig. 4:**
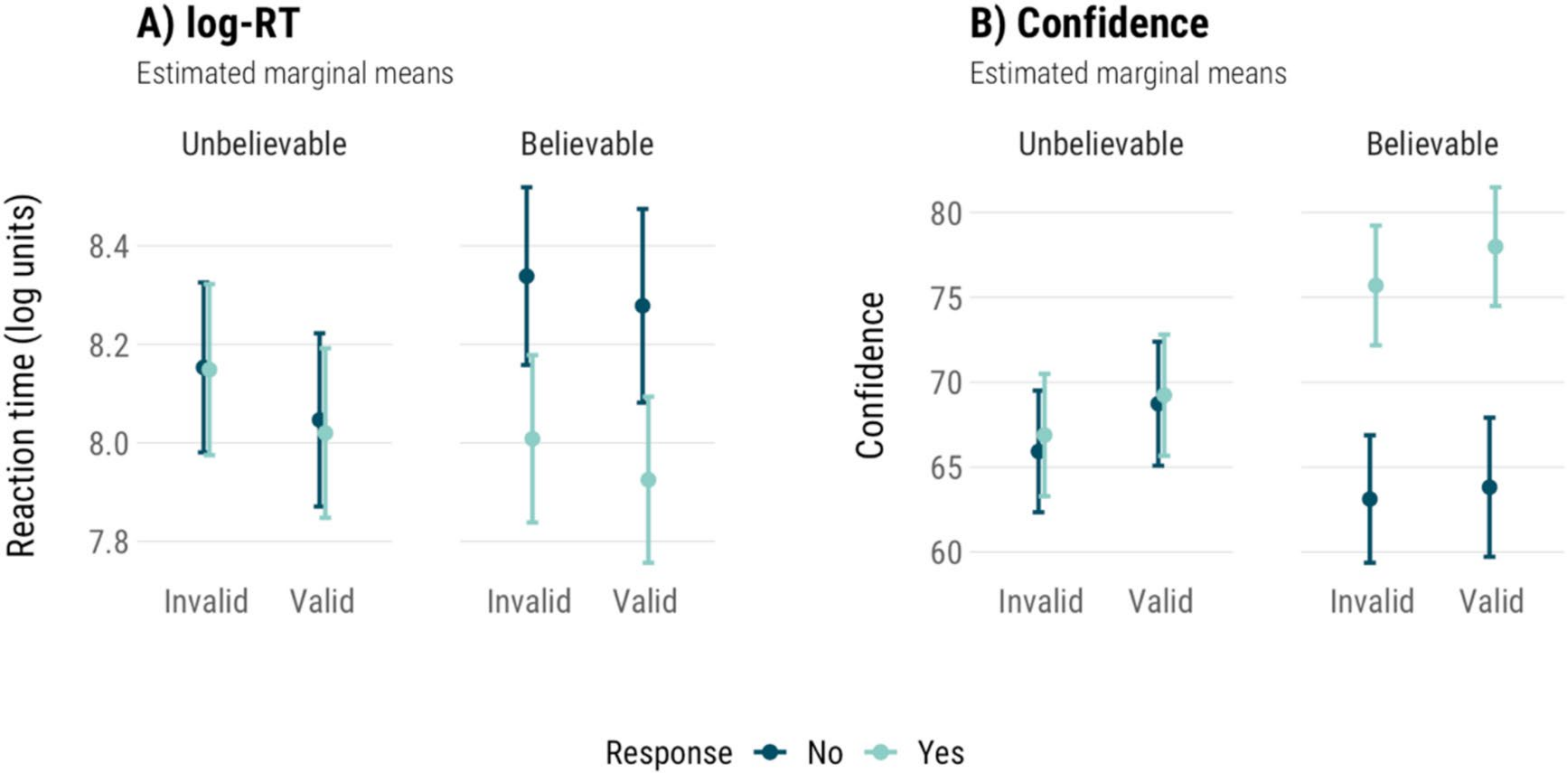
Estimated marginal means for log-transformed reaction times (log-RT) and confidence models. Error bars are 95% confidence intervals

If participants largely respond to AC and DA items as if they are valid, these items should be processed similarly to valid MP and MT items, with similar RT and confidence. We used response (‘yes’ – the conclusion is valid; ‘no’ – it is invalid), instead of accuracy, to compare effects on valid and invalid items more easily. The models for RT and confidence are summarised in Tables 5a and b, and the estimated marginal means are plotted in Fig. 4. Both models included response, validity, believability, and their interactions as fixed effects, with participant ID and item number as random effect.

**Table 5:** Predicting log-transformed reaction times (log-RT) and confidence based on response (yes, no), item validity (invalid, valid), and item believability (unbelievable, believable)

	Log-RT		Confidence	
Predictors	χ^2^	p-value	χ^2^	p-value
Response (No)	**45.57**	**<.001**	**152.14**	**<.001**
Validity (Invalid)	1.72	.19	**5.09**	**.02**
Believability (Unbelievable)	0.40	.53	**7.28**	**.007**
Response * Validity	0.21	.65	0.28	.59
Response * Believability	**39.82**	**<.001**	**132.57**	**<.001**
Validity * Believability	0.10	.75	0.37	.54
Response * Validity * Believability	0.00	.99	0.97	.33

Both dependent variables were predicted by response and an interaction between response and believability. For log-RT, these were the only significant predictors (*χ*^*2*^(1) = 45.57, *p* < .001; *χ*^*2*^(1) = 39.82, *p* < .001). On unbelievable items, ‘yes’ and ‘no’ responses were given with similar speed (*p* = .64), while on believable items ‘no’ responses were given more slowly than ‘yes’ responses (*OR* = 1.41, *p* < .001; also see Fig. 4). Confidence was predicted by response (*χ*^*2*^(1) = 152.14, *p* < .001), validity (*χ*^*2*^(1) = 5.09, *p* = .02), believability (*χ*^*2*^(1) = 7.28, *p* = .007), and an interaction between response and believability (*χ*^*2*^(1) = 132.57, *p* < .001). Participants were less confident when giving ‘no’ responses than ‘yes’ responses to believable items (*b* = −13.37, *p* < .001), but on unbelievable items there was no difference (*p* = .30). Valid and invalid items differed in confidence by only by 2% (69.9 vs. 67.9).

Validity was not a significant main effect for RT, and showed only a small (2%) significant difference in confidence judgements. There were no interactions with validity. RT and confidence were more strongly driven by conclusion believability and response type, and were only slightly affected by the validity of the item on one of the measures.

##### True and reverse detection effects

These data also tell us something about the apparent differences between true and ‘reverse’ detection effects. Traditional conflict detection measurement compares the following items, summarised in Table 6: ‘no’ responses to valid-unbelievable items versus ‘yes’ responses to valid-believable items; and ‘yes’ responses to invalid-believable items, versus ‘no’ responses to invalid-unbelievable ones. Decreased confidence and increased RT here are interpreted as evidence of conflict detection.

**Table 6:** Expected versus observed increases and decreases (from Fig. 4) in reaction time (log-RT) and confidence when comparing incorrect conflict and correct no-conflict items (the standard conflict detection measurement approach)

			Log-RT		Confidence	
	Incorrect conflict items	Correct no-conflict items	Expected	Observed	Expected	Observed
Valid	No-Unbelievable (no-VU)	Yes-Believable (yes-VB)	Increase	Increase	Decrease	Decrease
Invalid	Yes-Believable (yes-IB)	No-Unbelievable (no-IU)	Increase	Decrease	Decrease	Increase
Invalid (pseudo-valid)	No-Unbelievable (no-IU)	Yes-Believable (yes-IB)	Increase	Increase	Decrease	Decrease

As we see in the previous model, valid and invalid items were solved with similar RT and confidence. This means that for invalid items, the standard points of comparison (yes-IB vs. no-IU) create the ‘reverse’ CD effect. Using pairwise comparisons with the Bonferroni correction, we checked whether the expected conflict detection effects were still present in this data (‘Expected’ columns in Table 6). In terms of log-RT, there were no significant differences between ‘no’ responses on unbelievable items and ‘yes’ responses on believable ones (*p* = .65), in line with previous log-RT analyses. On invalid items, there were also no significant differences in log-RT (*p* = 0.50).

When we looked at confidence judgements, valid items showed an overall conflict detection effect in the expected direction (*b* = −9.25, *p* < .001), but for invalid items the difference was reversed (*b* = 9.77, *p* < .001). Comparing ‘no’ responses to unbelievable items and ‘yes’ responses to believable items dispels the reversed effect (*b* = −9.77).

**Fig. 5:**
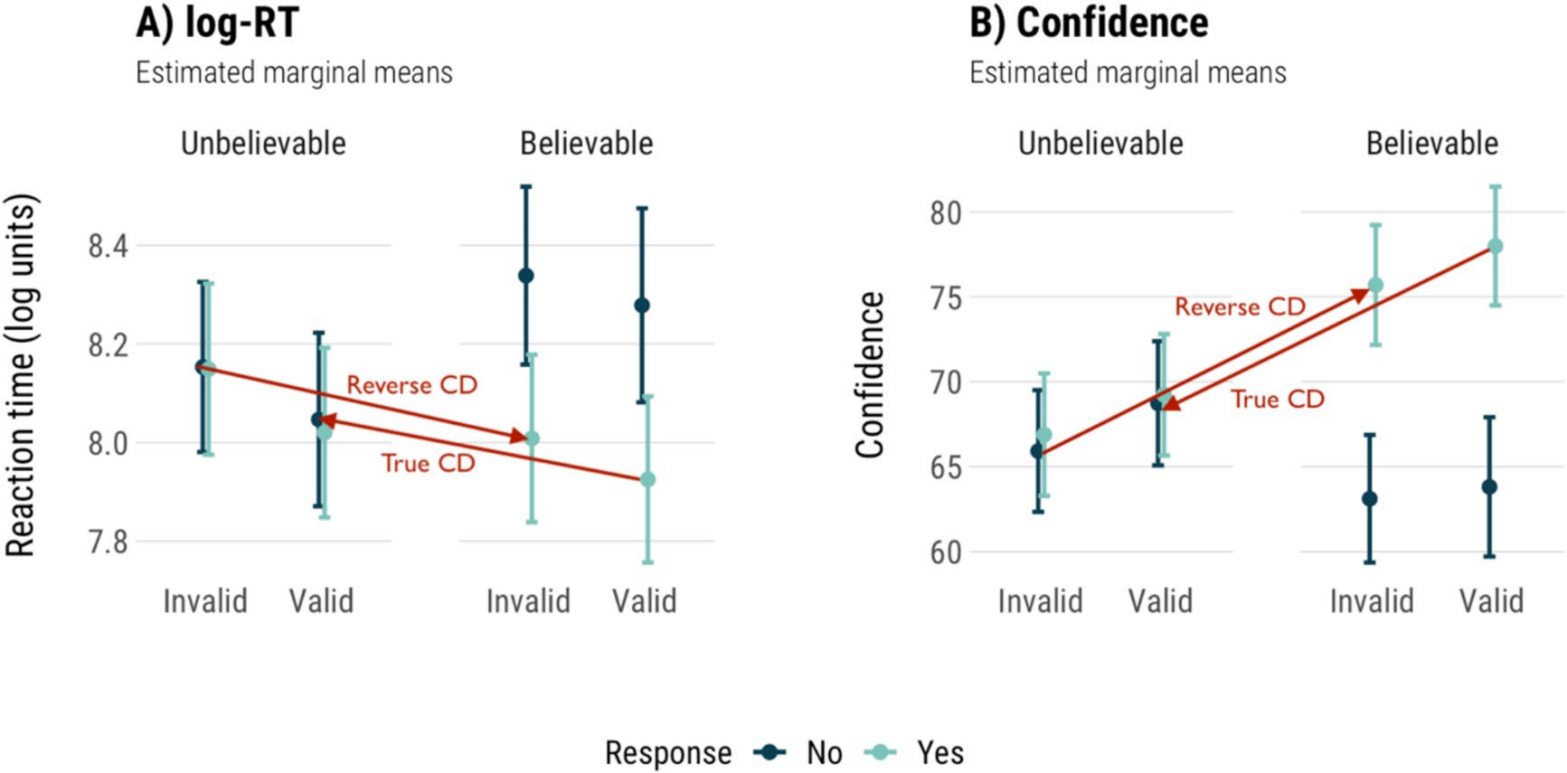
Experiment 1 models predicting (**a**) log-transformed reaction times (log-RT) and (**b**) confidence from response choice, validity, and believability. Red lines show which items are being compared when calculating conflict detection effects

Figure 5 shows the marginal means plot from the models of RT and confidence based on response, believability, and validity that we presented earlier, with overlaid red lines showing which items would be compared when calculating a conflict detection index. The lines indicating conflict effects should be crossed over – invalid-believable ‘yes’ response should be as high as the ‘no’ response in the log-RT plot, and as low as the ‘no’ response in the confidence plot. As Fig. 5 shows this is clearly not what is happening here. As there is no difference between valid and invalid items, comparing conflict and no-conflict versions of valid and invalid items gives almost identical results, just calculated in the opposite direction.

##### Summary

There were small or non-significant differences in confidence and RT between valid and invalid items, indicating that they are processed in a similar way. On unbelievable items, participants responded both ‘yes’ and ‘no’ with similar confidence and RT; on unbelievable items these measures diverged, so that people were slower and less confident when saying ‘no’, relative to when they say ‘yes’. ‘Reverse’ detection effects disappear if we consider invalid items to perceived as valid and adjust the points of comparison accordingly.

### Discussion

In Experiment 1 we have clearly demonstrated reverse detection effects on invalid AC and DA conditionals with an independent set of conditional syllogisms, with both RT and confidence ratings, confirming that this was not an anomalous result that Brisson et al. (2018) observed. Results from the speeded abstract task show that participants endorse both valid and invalid items as valid to a similar degree. Although we cannot be sure that the abstract task isolates logical intuition(s) perfectly, responding should reflect participants’ perceptions of the logical structure of the task more closely than the experimental items, because of the belief-neutral content and the time limit which would have limited the degree to which participants could engage in deliberation. The proportion of items endorsed as valid on the abstract task predicted how likely participants were to endorse a conclusion on invalid syllogisms in the main task. Participants only treated valid and invalid items differently when they had a strong intuition that invalid items are invalid, but such intuitions were not common.

RT and confidence did not reflect an overall sensitivity to belief-logic conflict either. Reaction time and confidence varied as a function of validity, believability, and response, but in a way that is not consistent with the idea that reasoners are intuitively sensitive to the logical status of invalid conditional syllogisms. Reaction times were less sensitive to the evaluated differences than confidence, with more variability obscuring the effects. Participants endorsed and rejected syllogism conclusions with the same speed and confidence on both valid and invalid unbelievable items, but ‘yes’ and ‘no’responses were clearly differentiated on believable items – however, there was still no meaningful difference between valid and invalid items. ‘Reverse’ detection effects appear to be a by-product of this more general pattern.

## Experiment 2

In Experiment 1, we have shown that participants tended to endorse the conclusions of abstract AC and DA conditionals under time pressure, and this predicted how often they endorsed the conclusions to our experimental AC and DA conditionals. These results are in line with the findings of Ghasemi et al. (2022) suggesting that participants tend to respond to these types of items through a matching heuristic rather than intuitive logic, which leads participants to treat AC and DA items as valid. This is further demonstrated by the ‘reverse’ detection effects on invalid syllogisms, which disappear if we consider these items to be perceived as valid, and compare incorrect conflict and correct no-conflict items based on this assumption.

In Experiment 2, we manipulate item content to increase endorsement rates on AC and DA conditionals. Our goal was to evaluate whether the reversed detection effects observed in Experiment 1 would be stronger on problems manipulated to increase the conclusion endorsement rate. Previous research has shown that the tendency to evaluate AC and DA arguments as valid is increased on more abstract items, which typically limit the availability of counterexamples (Evans et al., 2007; Evans et al., 2010; Newman et al., 2017).

Half the participants solved categorical syllogisms identical or very similar to those used by Brisson et al. (2018), which was the standard condition. The other participants received the same items, but with one of the terms in the premises replaced with a non-word, making premises belief-neutral, but keeping the same believable or unbelievable conclusion (neutral condition). Our principal hypotheses are detailed below:

- H1.1: Adding a non-word to the premises makes the item content more abstract, so AC and DA items in the neutral condition are expected to be endorsed as valid more often. This is expected to lead to greater ‘reverse’ detection effects, by the same mechanism as in the standard items, where no-conflict items are more likely to have subjective conflict, and conflict items more likely to have no conflict.
- H2.1: The speeded abstract task is expected to predict responses on neutral items more strongly than on standard items, because if the intuitive response to abstract-content items is to treat them as valid, then performance on the speeded and neutral items should be more closely aligned.
- H3.1: We expect that confidence and RT would vary between ‘yes’ and ‘no’ responses in the same way they varied in Experiment 1.

### Method

#### Participants

Participants were randomly assigned to either standard or neutral syllogism conditions, resulting in 73 participants in the standard, and 78 in neutral condition (151 participants total). Participants mostly identified as female (75%; age range 17–56 years, *M* = 22.6 years). Participants also completed a Stroop task and evaluated the believability of syllogism conclusions as part of another study not reported here.

#### Materials and procedure

##### Categorical syllogisms

Participants were randomly assigned to either standard item condition or neutral item condition. In both conditions, participants were asked to evaluate the logical validity of the conclusion of a set of categorical syllogisms (see Table 7 for example items). As in Experiment 1, we used 64 syllogisms of the same four types (MP, MT, AC, DA), so that half the items are valid, and half are invalid, and half are believable and the other half unbelievable. There were four practice trials, where participants were shown whether their response was correct or incorrect.

**Table 7:** Example standard and neutral categorical syllogisms

		Conflict			No conflict	
		Standard	Neutral		Standard	Neutral
MP	VU	All mammals can walk Whales are mammals Whales can walk	All hirks can walk Whales are hirks Whales can walk	VB	All plants need water Roses are plants Roses need water	All smolns need water Roses are smolns Roses need water
MT	VU	All mammals can walk Whales can’t walk Whales aren’t mammals	All mammals can chid Whales can’t chid Whales aren’t mammals	VB	All plants need water Rocks don’t need water Rocks are not plants	All plants need vak Rocks don’t need vak Rocks are not plants
AC	IB	All plants need water Roses need water Roses are plants	All plants need vak Roses need vak Roses are plants	IU	All whales are mammals Mammals can walk Whales can walk	All whales are hirks Hirks can walk Whales can walk
DA	IB	All plants need water Rocks aren’t plants Rocks don’t need water	All smolns need water Rocks aren’t smolns Rocks don’t need water	IU	All whales are mammals Whales can’t walk Mammals can’t walk	All broans are mammals Broans can’t walk Mammals can’t walk

V/I = valid, invalid, B/U = believable, unbelievable

Items were presented using the same step-by-step procedure as before, and were asked to provide a confidence judgement after each response. In the standard condition, participants were instructed to assume that the premises are true and to figure out if the conclusion necessarily follows. For neutral items, we also included the following: “Some words in the problems will be nonsense words. This is on purpose, just assume that whatever the premises say is true and decide if the conclusion is valid.”

##### Speeded abstract reasoning task

The same as in Experiment 1. Mean RT was 3.6 s (range 0–7 s), as compared to 5.4 s on the main task (range 0–708 s).

##### Exclusion criteria

First, we excluded all syllogism data if a participant’s accuracy on no-conflict MP problems was <75% (i.e., six out of eight items). This removed ten people in total, two from the standard condition and eight from neutral. We removed 25 log-RT data points trials that were unusually fast (< 3 SD from mean RT for a given participant), to filter out accidental or purposeful trial skips.

##### Conflict detection indexes

The same as in Experiment 1.

### Results

First, we looked at whether accuracy, RT, and confidence show the expected differences between conflict and no-conflict items, valid and invalid items, and standard and neutral conditions. For each model, we included conflict, validity, and condition as fixed effects, and participant ID and item number as random effects.

#### Accuracy

Accuracy was consistently lower on conflict relative to no-conflict items, in both the standard and neutral conditions. On standard items, there was a main effect of conflict (*χ*^*2*^(1) = 89.72, *p* < .001), validity (*χ*^*2*^(1) = 11.37, *p* < .001), but no significant interaction effect (*p* = .91). On neutral items, there was also a main effect of conflict (*χ*^*2*^(1) = 65.11, *p* < .001), validity (*χ*^*2*^(1) = 50.68, *p* < .001), and no significant interaction effect (*p* = .12).

Neutral items were designed to have lower counterexample availability, leading to lower accuracy on invalid neutral than invalid standard items. To check that this manipulation worked, we modelled accuracy by condition and validity. Overall, valid items were solved correctly more often than invalid items (*χ*^*2*^(1) = 18.05, *p* < .001, 0.82 vs. 0.57), but accuracy did not differ between conditions (*p* = .77), and there was no interaction effect (*p* = .15). Accuracy was lower on no-conflict invalid neutral items relative to no-conflict invalid standard items (64% vs. 81%; Table 8), but was the same for conflict items in both conditions (27%). This suggests that our manipulation had a limited effect on influencing accuracy, reducing accuracy on invalid no-conflict items, but not on invalid conflict ones.

**Table 8:** Mean accuracy, log-transformed reaction times (log-RT), and confidence judgements for standard and neutral items

			Accuracy (%)	Log-RT (SE)	Confidence (SE)
Standard	Valid	No-conflict	86	7.81 (0.03)	82 (0.60)
		Conflict	41	7.99 (0.03)	76 (0.70)
	Invalid	No-conflict	81	7.94 (0.03)	77 (0.65)
		Conflict	27	7.91 (0.03)	79 (0.65)
Neutral	Valid	No-conflict	79	8.07 (0.03)	77 (0.58)
		Conflict	59	8.16 (0.03)	73 (0.63)
	Invalid	No-conflict	64	8.34 (0.03)	69 (0.67)
		Conflict	27	8.11 (0.03)	74 (0.60)

#### Reaction time

In the standard condition, there was no main effect of conflict (*p* = .26), validity (*p* = .69), or their interaction (*p* = .10). In the neutral condition, there was no effect of conflict (*p* = .31) or validity (*p* = .12), but a significant interaction effect (*χ*^*2*^(1) = 5.35, *p* = .02). Pairwise contrasts showed significantly longer response times on no-conflict than conflict invalid syllogisms (*OR* = 1.03, *p* = .02), but not for valid ones (*p* = .34).

**Fig. 6:**
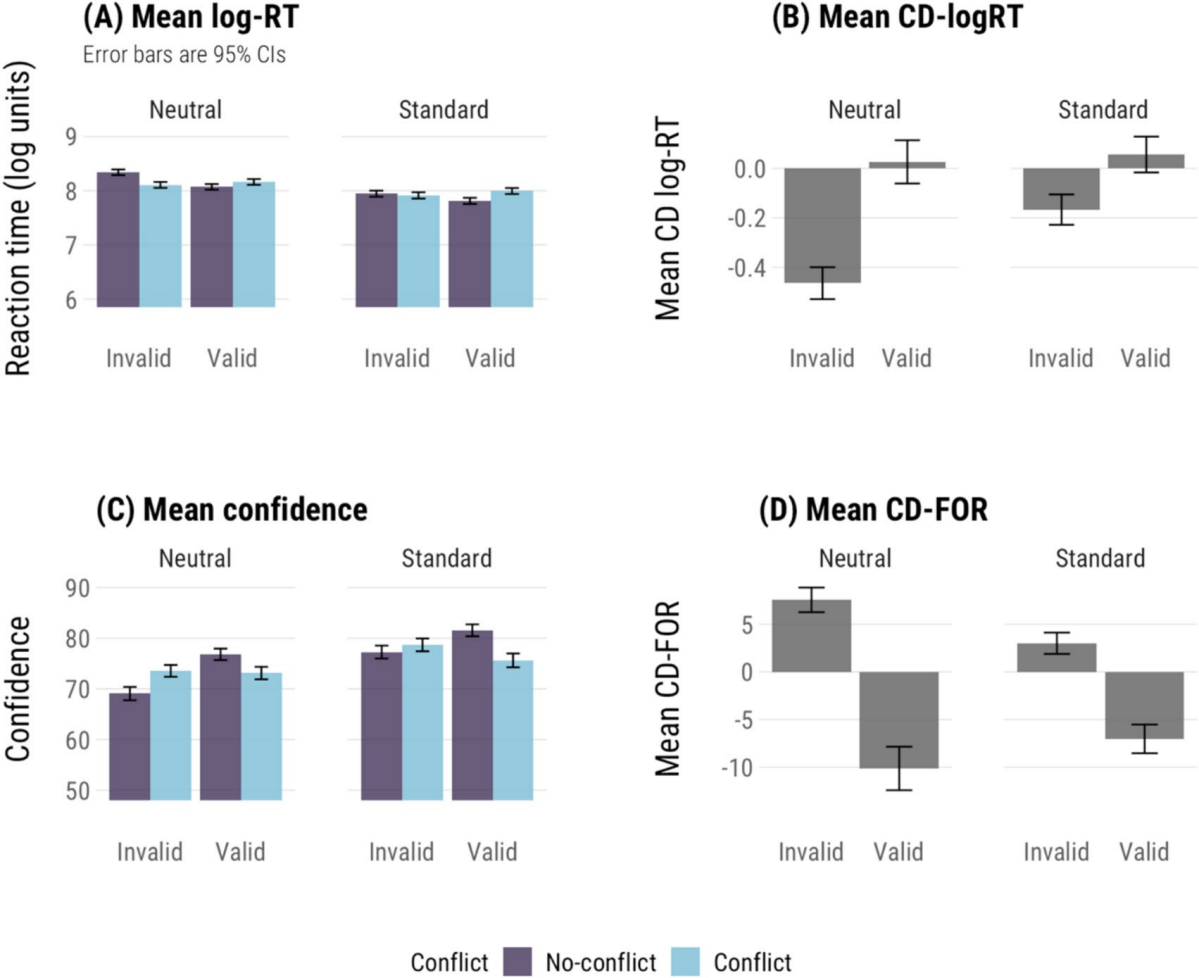
Mean log-transformed reaction times (log-RT) conflict detection index with log-transformed RTs (CD-logRT), confidence, and conflict detection index based on confidence judgements (CD-FOR). Error bars are 95% confidence intervals

We also tested whether validity and condition influenced CD-logRT indexes and whether there was a mean CD-logRT effect in the expected positive or the reverse negative direction. A mixed effects model with validity and condition as predictors had all significant effects. Valid items showed larger CD-logRT values than invalid items (*χ*^*2*^(1) = 17.03, *p* < .001, 0.14 vs. −0.17), standard items larger than neutral items (*χ*^*2*^(1) = 4.72, *p* = .03, 0.06 vs. −0.10), but there was no interaction between validity and condition (*p* = .13). Pairwise contrasts showed that while valid items had no significant differences in CD-logRT values between standard and neutral conditions (*p* = .55), invalid items had smaller CD-logRT values in the neutral condition (*OR* = 0.75, *p* = .01), indicating larger ‘reverse’ detection effects in this case (see Fig. 6b).

Mean CD-logRT values are shown in Fig. 6b. CD-logRT were not significantly different from zero on valid standard items (*p* = .13), but there was a significant reverse detection effect for invalid items (*t*(807) = −5.31, *p* < .001). In the neutral condition, valid items again showed no detection effect on average (*p* = .55), but invalid items showed a ‘reverse’ CD-logRT (*t*(768) = −13.98, *p* < .001). In sum, neutral invalid items tended to show larger reverse detection effects, in line with our predictions.

#### Confidence

Confidence ratings showed a similar pattern to log-RT in standard and neutral conditions (Fig. 6c). In the standard condition, there was a significant effect of conflict (*χ*^*2*^(1) = 8.69, *p* = .003), no effect of validity (*p* = .45), and a significant interaction effect (*χ*^*2*^(1) = 20.61, *p* < .001). There was no difference in confidence between no-conflict and conflict invalid items (*p* = .18), but valid no-conflict items were solved with more confidence than conflict items (*b* = 6.01, *p* < .001).

In the neutral condition, there was no significant effect of conflict (*p* = .63), a significant effect of validity (*χ*^*2*^(1) = 18.34, *p* < .001), and a significant interaction effect (*χ*^*2*^(1) = 21.86, *p* < .001). No-conflict invalid items were solved with less confidence than conflict invalid items (*b* = −4.46, *p* < .001). On valid syllogisms, no-conflict items were solved with more confidence than conflict items (*b* = 3.68, *p* = .002). Overall, participants’ confidence ratings were more sensitive to conflict in the neutral than in standard condition.

We again tested how CD-FOR values were influenced by item validity and condition, as well as whether there were greater ‘reverse’ detection effects on neutral invalid items than standard invalid items (i.e., more positive CD-FOR values). CD-FOR values were significantly lower for valid than invalid items (*χ*^*2*^(1) = 59.48, *p* < .001, −6.97 vs. 1.54), and lower in the standard than neutral condition (*χ*^*2*^(1) = 7.39, *p* = .007, −4.35 vs. −1.06). The interaction effect was not significant (*p* = .49). Mean CD-FOR effects were significantly different from zero for valid standard items (*t*(660) = −9.16, *p* < .001), and for invalid standard items, in the reverse direction (*t*(808) = 5.23, *p* < .001). In the neutral condition, there was again the expected detection effect, showing negative mean CD-FOR for valid items (*t*(378) = −8.70, *p* < .001), and the reverse effect with positive mean CD-FOR for invalid items (*t*(771) = 11.43, *p* < .001). Confidence judgements produced greater ‘reverse’ detection effects on invalid neutral than invalid standard items, but the difference was not significant, as indicated by the absent interaction effect in the model above.

#### Summary

Accuracy and confidence judgements were both influenced by belief-logic conflict, but RT showed no overall conflict effects. Neutral items were designed to be more abstract, leading to a higher conclusion endorsement rate on invalid syllogisms, so we expected lower accuracy, larger negative CD-logRT, and larger positive CD-FOR values for invalid neutral than invalid standard syllogisms. Standard and neutral conditions did not significantly differ by accuracy overall, although no-conflict invalid items were solved less accurately in the neutral than standard condition, which is the direction we expected. We observed the expected larger negative CD-logRT on invalid neutral than invalid standard items, but not the more positive CD-FOR values, which differed by validity and by condition, but did not interact. Nonetheless, both CD-logRT and CD-FOR showed ‘reverse’ detection effects on invalid items in both conditions that were significantly different from zero, replicating the key findings from Experiment 1.

#### What is the intuitive response to AC and DA conditional syllogisms?

Here we repeat the analyses from Experiment 1, looking at how participants responded on a speeded abstract reasoning task and the relationship between the tendency to endorse syllogisms as valid and the proportion of endorsed conclusions on the abstract task. ‘Yes’ responses indicate that the participant considered the conclusion valid, and ‘no’ if they considered it not valid.

In this sample, participants endorsed the conclusions of valid and invalid items on the speeded abstract task at a similar rate: participants endorsed the conclusion of 63% of valid and 62% of invalid items on average. Endorsement rates did not differ by validity (*p* = 0.7). Under time pressure, participants tended to treat valid and invalid items the same way, responding as if they judged abstract AC and DA forms as valid.

**Fig. 7:**
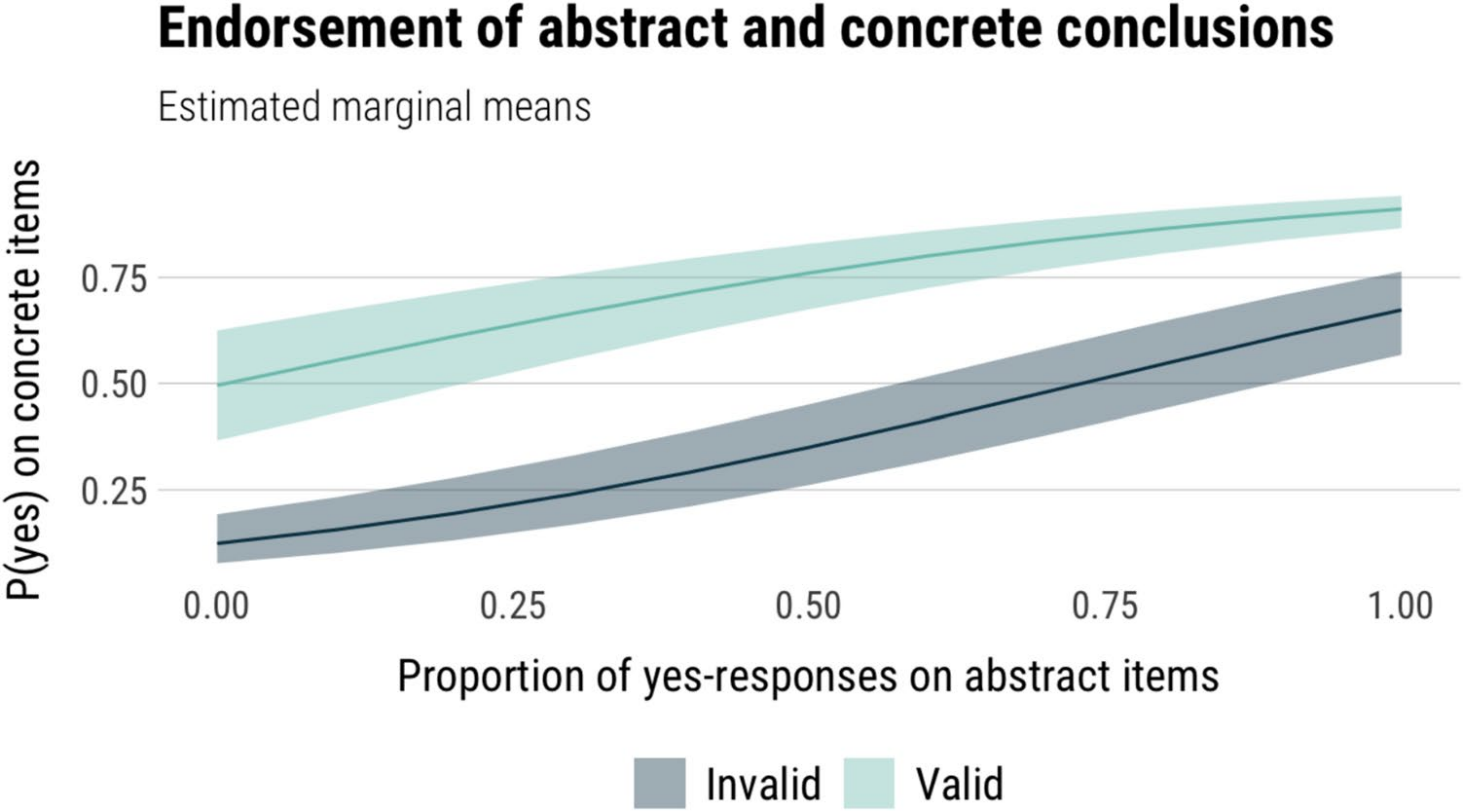
Estimated marginal means of the model predicting yes-responses on invalid syllogisms from yes-responses on an abstract syllogism task on standard, and neutral items

*Does logical intuition predict responses?* Next, we modelled how rates of conclusion endorsements on abstract syllogisms on the speeded task influenced conclusion endorsements in the main task. We modelled whether ‘yes’ responses on the main task were predicted by ‘yes’ responses on the speeded items and item validity. We did not include condition as a factor, since our earlier analyses showed that while there were differences in RT and confidence between standard and neutral items, these did not translate to differences in responses. The estimated marginal means of the model are plotted in Fig. 7.

There was a significant effect of abstract item conclusion endorsement on main task conclusion endorsement (*χ*^*2*^(1) = 138.68, p < .001), and a main effect of validity (*χ*^*2*^(1) = 32.61, *p* < .001), with no significant interaction between them (*p* = .219). We used pairwise contrasts to confirm the relationship between main and abstract conclusion endorsements separately for valid and invalid items, as we were primarily interested in this relationship on invalid arguments. Participants were more likely to endorse the main item conclusion as valid when they have endorsed more of the abstract items as valid, on both valid (*OR* = 10.5, *p* < .001) and invalid items (*OR* = 14.6, *p* < .001).

#### Relationship between reaction time (RT), confidence, and the chosen response

**Fig. 8:**
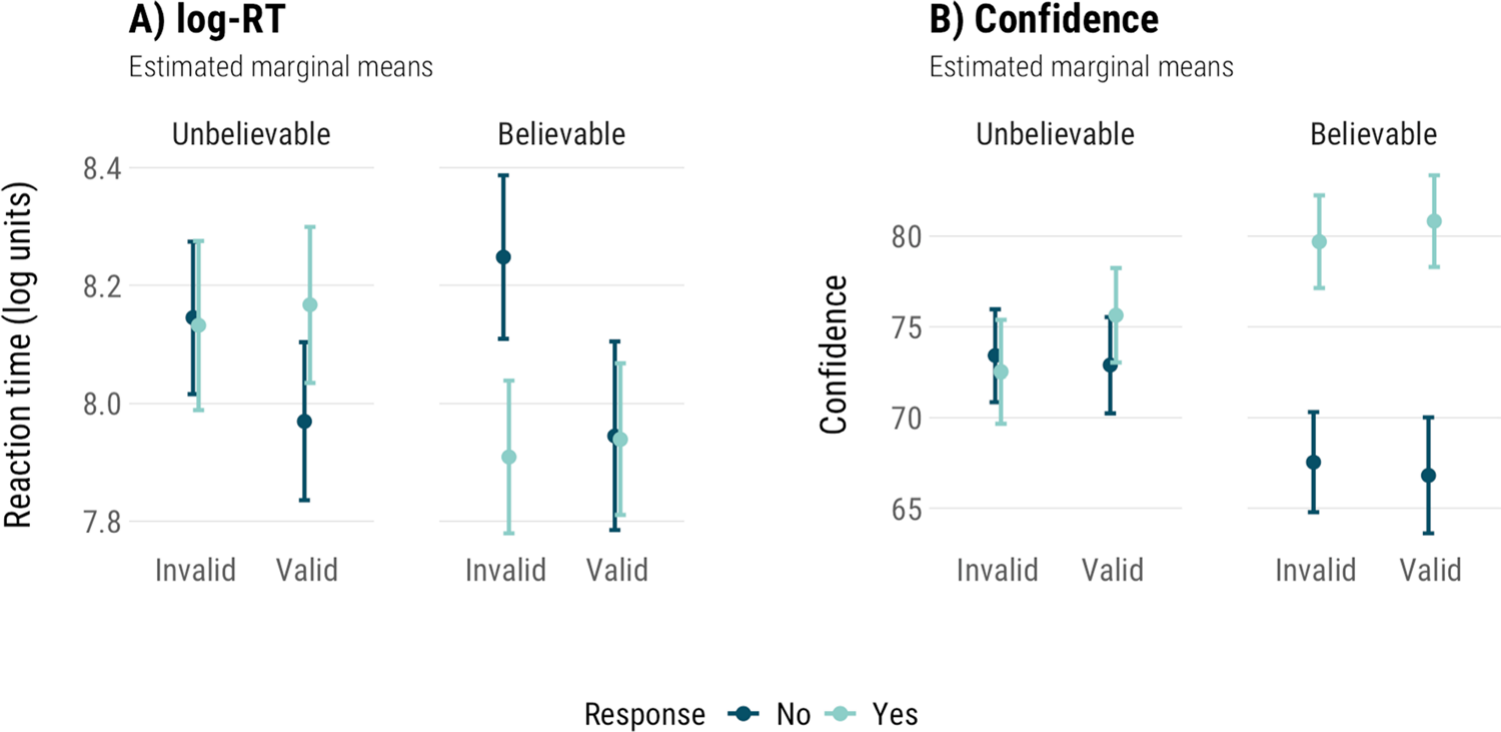
Estimated marginal means for (**a**) log-transformed reaction times (log-RT) and (**b**) confidence models, with both standard and neutral items. Error bars are 95% confidence intervals

As in Experiment 1, we explored the general relationship between process measures (RT, confidence) and how participants responded to different types of items. We modelled RT and confidence as predicted by response, validity, and believability. Analyses were collapsed across condition, as we have no predictions about condition differences here that we have not analysed earlier. The results are summarised in Table 9, and marginal means of these models are shown in Fig. 8.

**Table 9:** Predicting log-transformed reaction times (log-RT) and confidence based on response (yes, no), item validity (invalid, valid), and item believability (unbelievable, believable). Includes both standard and neutral items

	Log-RT		Confidence	
Predictors	χ^2^	p-value	χ^2^	p-value
Response (No)	3.19	.07	**223.34**	**<.001**
Validity (Invalid)	**4.21**	**.04**	1.78	.18
Believability (Unbelievable)	3.42	.06	0.04	.84
Response * Validity	**37.15**	**<.001**	**8.80**	**.003**
Response * Believability	**35.04**	**<.001**	**168.56**	**<.001**
Validity * Believability	0.44	.51	0.93	.33
Response * Validity * Believability	2.03	.15	0.98	.32

Reaction time was predicted by validity (*χ*^*2*^(1) = 4.21, *p* = .04), and interactions between response and validity (*χ*^*2*^(1) = 37.15, *p* < .001), and response and believability (*χ*^*2*^(1) = 35.04, *p* < .001). The effects of response, believability, interaction between validity and believability, and the three-way interaction were not significant (*p*s > .06). Pairwise contrasts showed that ‘no’ responses were given faster than ‘yes’ responses on valid items (*OR* = 0.91, *p* = .004), but on invalid items, ‘no’ responses were given slower (*OR* = 1.19, *p* < .001). In case of the believability interaction, ‘no’ responses were given slower than ‘yes’ responses on believable items (*OR* = 1.19, *p* < .001), and faster than ‘yes’ responses (*OR* = 0.92, *p* = .001) on unbelievable items.

Looking at Fig. 8a, it appears that ‘yes’ responses to valid and invalid items are given with the same speed within each believability level, but ‘no’ responses are given with different speeds on valid and invalid items. Participants were faster to reject valid items of either believability, and slower to reject invalid items.

Confidence was predicted by the response (*χ*^*2*^(1) = 223.34, *p* < .001), and the interactions between response and validity (*χ*^*2*^(1) = 8.80, *p* = .003), and response and believability (*χ*^*2*^(1) = 168.56, *p* < .001). There was no main validity effect. ‘Yes’ and ‘no’ responses were given with similar confidence on unbelievable items (*p* = .12), and ‘yes’ responses were given with more confidence than ‘no’ responses on believable items (*b* = −13.08, *p* < .001). ‘Yes’ responses were given with more confidence than ‘no’ responses on both valid (*b* = 8.38, *p* < .001) and invalid items (*b* = 5.63, *p* < .001).

##### True and ‘reverse’ detection effects

Next, we demonstrate that the ‘reverse’ detection effects on invalid items disappear if we treat them as pseudo-valid when evaluating whether conflict detection was present. Here we use the same points of comparison and predictions of the differences in RT and confidence that were outlined in Table 6. Briefly, we expect both valid and pseudo-valid items to show an increase in RT and a decrease in confidence on ‘no’ responses to unbelievable items relative to ‘yes’ responses to believable items (incorrectly-solved conflict items vs. correct no-conflict items). We use pairwise contrasts from the models above to demonstrate these differences.

In terms of RT, valid items require a comparison between no-VU and yes-VB items, which was not significant (*p* = 0.65). The standard points of comparison for invalid items are between yes-IB items and no-IU items, again showing no significant difference (*p* = 0.50). Comparing confidence between no-VU and yes-VB items, we saw a significant decrease in confidence (*b* = −7.95, *p* < .001), indicating a true conflict detection effect on valid items. Treating invalid items as invalid, we observed a positive difference between yes-IB and no-IU items (*b* = 6.29, *p* < .001), indicating a ‘reverse’ detection effect. If we treat these items as pseudo-valid and instead compare no-IU and yes-IB items, then we see the expected decrease in confidence (*b* = −6.29) between incorrectly solved ‘conflict’ and correctly solved ‘no-conflict’ items.

### Discussion

In Experiment 2, we evaluated whether more abstract (neutral) items would show greater endorsement rates on AC and DA inferences and lead participants to treat them as pseudo-valid more strongly than standard categorical syllogisms. We examined whether this would lead to larger ‘reverse’ detection effects and a stronger association with performance on the speeded abstract task. Participants solved neutral items with the same accuracy as standard items, overall. However, neutral items still showed larger ‘reverse’ detection effects than standard items. CD-logRT values were smaller on neutral invalid items than standard invalid items, reflecting larger ‘reverse’ detection effects on neutral items. CD-FOR values were overall larger in the neutral condition, but this reflected both a larger ‘reverse’ detection effect for invalid items and a larger true detection effect for valid items, relative to the standard condition. Changes in conflict detection effects between standard and neutral conditions demonstrate that despite non-significant differences in accuracy, the processing of these items was affected by the experimental manipulations.

Valid and invalid items on the speeded abstract task were endorsed as valid at the same rate (62–63%), indicating that intuitively, participants tend to treat both as valid inferences. This is consistent with the findings of Ghasemi et al. (2022) suggesting that responses on AC and DA conditionals are to a large extent driven by a matching heuristic, rather than automated learned logical skills (whether mindware, Stanovich et al., 2016, or intuitive logic). Conclusion endorsements on the speeded task predicted conclusion endorsements on the main task, and there was an overall difference in endorsement rates between valid and invalid items – contrary to our hypothesis that valid and invalid items are processed the same way. However, the overall pattern of findings suggests that most participants respond similarly to valid and invalid syllogisms, and inconsistent with the idea of a universally available logical intuition on invalid items.

Finally, we predicted that RT and confidence would not differ between valid and invalid items, when we also account for the believability of the conclusion and whether or not the conclusion was endorsed or rejected. Valid and invalid items were generally answered with the same speed and confidence – with the exception of frequentist analyses of RT (but not the Bayesian analyses). The general pattern is similar to what we observed in Experiment 1: process measures are similar when rejecting or accepting conclusions to unbelievable items, but different when endorsing or rejecting a believable conclusion, with little-to-no impact of validity. As a result, ‘reverse’ detection effects disappear if we consider invalid items as valid.

## General discussion

We begin our general discussion by returning to the conditional syllogism that we presented at the beginning of the article:

*All flowers need water.*

*Roses need water.*

*Therefore, roses are flowers*.

Arguments of this kind are ubiquitous in studies of belief bias in human reasoning (see, e.g., Markovits & Nantel, 1989) and problems of this kind are included as part of the comprehensive assessment of rational thinking (Stanovich et al., 2016, p.340). Structurally similar syllogisms have since been used in a number of dual processing and conflict detection studies (Bago & De Neys, 2017; Brisson et al., 2018; De Neys et al., 2010; De Neys & Franssens, 2009; Stanovich & West, 1998; West et al., 2008). In these problem sets, AC and/or DA items often make up half the problems and so have a substantial influence on the overall results. Typically, analyses are collapsed across item types, and conflict detection indices are calculated as an overall difference in RT or confidence between incorrectly-solved conflict and correctly-solved no-conflict items. Any overall increase in RT or decrease in confidence are interpreted as evidence of sensitivity to belief-logic conflict in syllogisms, as a precursor to the ability to deliberate and reach the correct response. In contrast, conflict detection effects close to zero, with either RT or confidence, indicate no difference in processing of conflict and no-conflict items, and therefore no conflict detection.

These comparisons are based on an objective definition of belief-logic conflict, based on the normative status of the item. Our findings suggest that participants tend to treat AC and DA conditionals as if they are valid, which creates a mismatch between objective and subjective conflict in these problems. In the case of AC and DA inferences, researchers are essentially looking for evidence of conflict detection on the opposite set of items. If participants respond as if AC and DA are valid, then conflict items with believable conclusions do not have any subjective conflict to be detected. Conversely, normatively no-conflict invalid items with unbelievable conclusions would involve subjective conflict between the intuition that they are valid and the unbelievable conclusion, leading some participants to detect conflict, engage in reflective thinking, and reach the normatively correct conclusion.

Participants endorsed the conclusions to valid and invalid abstract syllogisms in both experiments at the same rate when responding under time pressure, which is thought to reveal the primary intuitive response to a task. Participants were more likely to endorse a syllogism’s conclusion as valid on the main task if they endorsed more speeded abstract items as valid. Further, we modelled RT and confidence as predicted by whether or not participants endorsed the conclusion as valid, and actual item validity and believability. Participants endorsed and rejected unbelievable conclusions with the same speed and confidence. They took longer and were less certain when rejecting believable conclusions, and were fast and sure to accept them – but there were no differences between valid and invalid items.

Because valid and invalid items are processed the same way, but conflict detection effects are calculated based on where conflict is objectively expected to happen, AC and DA items show ‘reverse’ detection effects consistent with select findings previously observed in the literature (Brisson et al., 2018). We found ‘reverse’ detection effects both in conditional (Experiment 1) and categorical invalid syllogisms (Experiment 2), with both RT and confidence.

In Experiment 2 we investigated whether ‘reverse’ detection effects can be increased by manipulating a set of items to be more abstract, which we assume provides less context to generate counter-examples from, leading participants to endorse the conclusion as valid more readily. We did not find the expected decrease in accuracy on neutral items overall, but there were still larger ‘reverse’ detection effects on neutral items. This discrepancy highlights that there may be relatively subtle differences between types of reasoning tasks that are reflected in process measures, but do not translate to changes in accuracy. These effects disappeared when detection effects were calculated based on subjective conflict. In future work more direct manipulation of counter-examples for invalid inferences and disablers for valid inferences would be useful in determining whether such manipulations reduce the rates at which intuitive inferences are drawn and hence conflict detected.

While we explain reverse detection effects on AC and DA items by a mismatch in subjective and objective conflict, one alternative explanation is that people evaluate unbelievable conclusions more slowly than believable conclusions, and unbelievable conclusions are a stronger competitor to logical inference (Brisson et al., 2018). Brisson and colleagues initially analysed valid and invalid items separately, meaning that if people are only sensitive to believability and not to conflict, valid items would appear to have a conflict detection effect – valid-unbelievable items would take longer than valid-believable items because unbelievable conclusions take longer to process. On invalid items, this would lead to a reversed detection effect, if invalid-unbelievable (no-conflict items) are processed more slowly than invalid-believable (conflict items). They tested for this confound between conflict and believability by comparing valid and invalid items at the same believability level (VB vs. IB, and VU vs. IU), and found that in these analyses, both believability and conflict had an effect.

Our data do not agree with their findings, as our more detailed analyses modelling RT and confidence from response, validity, and believability show that there are no stable differences between valid and invalid items, and the only major difference in RT and confidence are between ‘yes’ and ‘no’ responses to unbelievable items. Together with analyses of the speeded abstract task showing that participants generally do not have a logical intuition that invalid items are invalid, we conclude that the main driver of ‘reverse’ detection effects is the absence of validity effects and the resulting mismatch between subjective and objective conflict.

Based on previous work (Ghasemi et al., 2022), we predicted ‘reverse’ detection effects based on the assumption that responses on AC and DA items are at least partially driven by the matching heuristic, leading participants to endorse these items as valid. This account would suggest that ‘logical intuitions’ are not arising through a process of reasoning, instead depending upon a heuristic process that is sensitive to superficial structural features. As discussed earlier, MMT would claim that a reasoning process is engaged, but it is incomplete; reasoners construct an initial model of the premises that corresponds to just the ‘P and Q’ cases and fail to flesh out the full set of models of the conditional. Whilst this explanation can account for the tendency to endorse AC arguments and the associated reverse detection effects on these problems, it has more difficulty accounting for the similar findings that we observed on DA arguments. In this case, the initial ‘PQ’ model would need to be fleshed out in the following way to represent the not-P, not-Q contingency:

P: Q
not-P: not-Q

**Fig. 9:**
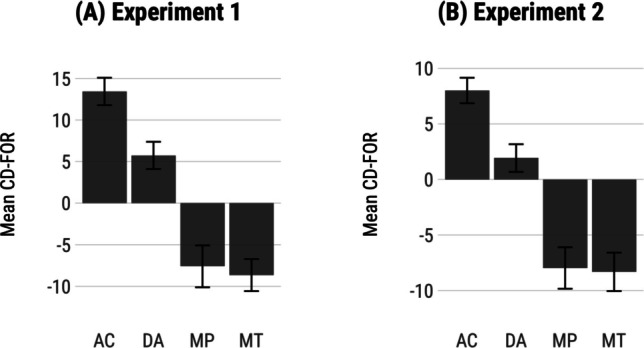
Mean conflict detection effects (CD-FOR) and 95% confidence intervals for each of the four syllogism types, in (**a**) Experiment 1 and (**b**) Experiment 2

Presumably this would require additional processing effort and hence one might expect ‘logical intuition’ effects to be weaker on these arguments. Looking at the mean conflict detection effects for each of the four syllogism types (Fig. 9), we can see that there is indeed a smaller, but reliable, conflict detection effect on DA items. Although this is partially consistent with this account, according to the model theory, MT also requires the full set of models to be fleshed out, but here MP and MT items clearly produce the same conflict detection effects. Therefore, mental model theory does not fully explain our findings.

An alternative explanation of high endorsement rates in these syllogisms is that they are interpreted as biconditionals, in which case it could be said that a logical intuition is involved after all, although not the expected one. Our manipulation of content, which we assume influences counterexample availability, could be said to also invite a biconditional reading of the major premise, leading to higher endorsement rates than standard items (although in our case there was no increase in endorsement rates, only in ‘reverse’ detection effects). Ghasemi et al. (2022) argue against biconditional reading of AC and DA items being the primary explanation of the high conclusion acceptance rates, based both on their analyses and prior work demonstrating the flexibility of acceptance rates depending on counterexample availability (e.g., Newman et al., 2017; Thompson, 1994). People are sensitive to background knowledge and additional information when evaluating these items as valid or invalid (e.g., Byrne, 1989; Oaksford et al., 2000). This suggests that a biconditional reading is not the default or fixed way to interpret them, so it is arguably not a logical intuition per se, or only as far as people reliably make that inference once background knowledge is taken into account. It could be argued from our data that because ‘reverse’ detection effects are present in both standard and neutral items in Experiment 2, even if biconditionality is involved, it is not the only factor driving the effect. It is possible that neutral content is not triggering a biconditional reading, but rather elicits a stronger matching heuristic, because the structural features appear more prominent and semantic features less salient because of the non-word term.

This ambivalence about whether or not a biconditional reading is involved points out a weakness in the concept of logical intuitions and the assumption of their involvement in conflict detection. Logical intuitions are defined by the fast normatively correct response they elicit. However, it is also commonly acknowledged that people vary in the degree of automaticity of various skills and knowledge related to logic and probability principles relevant to reasoning (De Neys, 2023; Stanovich et al., 2016). Further, it has been suggested that logical intuitions more generally arise from intuitive adherence to probabilistic rules, rather than classical logic (Ghasemi et al., 2023). There is now extensive accumulated evidence for people deviating from classical logic, but making inferences in line with Bayesian probability theory (Oaksford & Chater, 2020). The probabilistic paradigm emphasises the role of background knowledge and content in guiding reasoning, as well as the role of reasoning in social contexts, such as argumentation and persuasion. Oaksford and Chater (2020) present an extensive set of cases where strict adherence to logical norms without considering argument content produces non-sensical conclusions, and point out that the traditional approach to logical inference does not account for learning and belief change, or reasoning in a conversational context. In particular, it has been shown that invalid conditional inferences, such as AC, are not valid in a formal sense, but are nonetheless probabilistically strong when certain assumptions hold (e.g., Oaksford et al., 2000).

Variations in automaticity of logical intuitions and the alignment of reasoning with probabilistic principles challenges the view of people as ‘intuitive logicians’ that was originally aligned with the idea of intuitive logic (De Neys, 2012) and has been one of the key elements of DPT 2.0 (De Neys, 2018). If an intuition can be more or less automatic, and logical reasoning itself is more closely aligned with probabilistic principles and heavily influenced by background knowledge, then the response is determined by many more factors than the abstract logical structure of the problem, even when it is a fast normative response. In this case, it seems that a ‘logical intuition’ can appear and disappear depending on the person and the item, just like the ‘reverse’ detection effects were demonstrated to appear and disappear depending on what items were considered to elicit conflict. From this probabilistic point of view, conflict detection is more about coherence of various pieces of background knowledge and other problem information, rather than the specific conflict between conclusion believability and intuitions about validity. Absence of the expected logical intuitions and the subsequent misalignment of subjective and objective conflict might explain why some studies fail to find conflict detection effects in syllogisms (Swan et al., 2018), and possibly the absence of detection effects in other tasks (Ferreira et al., 2016; Travers et al., 2016) or for the subsets of participants (Frey et al., 2018; Mevel et al., 2015; Pennycook et al., 2015).

Such views fit with the latest uncertainty monitoring account of conflict detection (De Neys, 2023), which allows many intuitions to be produced in response to a problem, rather than the belief-based and logic-based intuitions that earlier models have focused on (De Neys & Pennycook, 2019), and does not require any of the intuitions to be logical per se. Deliberation is proposed to be triggered by the overall uncertainty parameter generated from the sum of these intuitions, rather than by the specific belief-logic conflict. However, the current approach to measuring conflict detection is not equipped to handle this more general view. As we demonstrate in this study, under some circumstances it can generate diametrically opposing results because of the strong mismatch between objective and subjective conflict.

Understanding how conflict detection works is a key step towards understanding when and how people are able to think beyond heuristics and biases. Syllogisms and other conflict tasks are also used to assess individual differences in rationality or deliberative thinking (e.g., Comprehensive Assessment of Rational Thinking; Stanovich et al., 2016). Understanding the mechanics behind these tasks has implications both for research and applied settings, as these assessments are used to study individual differences associated with the ability to solve heuristics and biases tasks (e.g., Toplak et al., 2016; Toplak & Flora, 2020) and measure the effectiveness of critical thinking and debiasing training (e.g., Janssen et al., 2019; Peppen et al., 2021). Designing training programs in particular depends on adequate understanding of where exactly the difficulties arise from.

At least with syllogisms, the discrepancy between subjective and objective conflict is an obstacle to using detection indices in their current form. While this makes studying conflict detection in syllogisms more difficult, it makes them a good testing ground for anticipating the kind of difficulties one may encounter when trying to gauge conflict detection effects in more naturalistic settings with more ecologically valid materials. To understand how people reason with syllogisms, it would be more informative to consider what factors influence RT or confidence in general, and how this relates to accuracy, or changing one’s mind in the two-response paradigm, rather than using a detection index that is grounded in assumptions about item categories that are based upon inappropriate normative principles. Alternatively, additional measures should be included to gauge the actual intuitive responses produced by each participant (De Neys, 2023). The process of developing measurement methods and elaborating on our understanding of constructs being studied go hand-in-hand (Bringmann et al., 2022). How we measure conflict detection should reflect our current understanding of the construct, if we want to develop this understanding further.

The ability to detect conflict between incompatible responses is thought to be a key element that enables people to engage in reflective thinking and override their biases or errors. Previous work defined conflict as the product of a logical intuition and a similarly intuitive belief-based response. It assumed that logical intuitions are common for certain simple problems, including the types of syllogisms we considered, and that they will produce normatively correct responses when not outweighed by conclusion believability. Based on previous work demonstrating that reasoners tend to endorse AC and DA as valid, a response misaligned with formal propositional logic, we suggested that this will be reflected in conflict detection measures on these items. We found, in line with previous work, that AC and DA items are commonly responded to as if they as valid, as shown by the speeded abstract task. Perceiving these items as valid leads to a discrepancy between items where experimenters expect conflict to occur, and where conflict is subjectively present to participants. This produces ‘reverse’ detection effects, which disappear if this is accounted for. This contradicts previous work suggesting that logical intuitions are necessary for conflict detection, and suggests that a more general conflict between different automatic responses of any kind is sufficient.

## Supplementary Information

Below is the link to the electronic supplementary material.

Supplementary file1 (DOCX 21 KB)

## Data Availability

Online materials, analysis code, and data are accessible via the Open Science Framework (10.17605/OSF.IO/MSDN2).

## Declarations

### Preregistration

No experiments were preregistered.
