# Supplementary material for: Conflict detection with invalid inferences: All heuristics, no logic
Source: Mem Cognit. 2025 Apr 17;53(7):2255–78. doi: 10.3758/s13421-025-01709-w (PMC12589286; doi:10.3758/s13421-025-01709-w)
Supplement: Supplementary file 1 — Supplementary file1 (DOCX 21 KB) [file 13421_2025_1709_MOESM1_ESM.docx]

# Supplementary analyses for the paper:

# “Conflict Detection With Invalid Inferences: All Heuristics, No Logic”

Here we include Bayesian analyses supplementing the key models in Experiments 1 and 2. We use the Bayesian parameter estimation approach (Kruschke & Liddell, 2018). It is more suitable to evaluating the degree of evidence in support of or against the effect than frequentist methods, which we use to gauge the degree to which absent effects (e.g., of validity) are likely to be truly absent.

We report the posterior estimate as either odds ratios or beta-coefficient as appropriate, and the highest density interval (HDI), which contains 95% of credible parameter estimates. We also calculate the proportion of credible values (CV), which counts how many of the estimates within the HDI fall on the same side of zero as the main reported estimate. The effect is more likely to be real if the CV is closer to 1.0 and the HDI does not include zero (or *OR* = 1.00) as a possible estimate value. The code for all the analyses is available on the OSF page for this project (https://osf.io/msdn2/).

**Experiment 1**

**(2.2.1) What is the intuitive response to AC and DA conditional syllogisms?**

First, we compared whether participants endorsed the conclusions of abstract valid and invalid items with the same probability. The effect of validity here is not credible, because the highest density interval includes the null effect, i.e., *OR* = 1.00, and a small proportion of the credible values includes null or opposite effects (*OR* = 0.74, *HDI* = 0.52, 1.07; *CV* = 0.94).

Second, we again ran a model testing if validity of the item and the proportion of conclusions endorsed as valid on the abstract task (calculated for valid and invalid items separately) predicts responses on the main task. We found a strong effect of abstract task endorsements, with all credible values being within the HDI (*OR* = 5.32, *HDI* = 3.26, 8.85; *CV* = 1.0). There was also a highly likely main effect of validity, indicating that overall invalid items are endorsed less than valid items on the main task (*OR* = 0.49, *HDI* = 0.32, 0.76; *CV* = 0.99). The interaction between validity and abstract task performance is also credible (*OR* = 1.54, *HDI* = 1.12, 2.11; *CV* = 0.99). Pairwise contrasts show that there is a positive relationship between the proportion of conclusions endorsed as valid on abstract items and the probability of endorsing the main task conclusion as valid, for both valid (*OR* = 3.47, *HDI* = 1.60, 6.25) and invalid (*OR* = 8.18, *HDI* = 4.46, 12.88) main task items. The Bayesian analyses replicate the frequentist analyses.

**(2.2.2) Relationship between reaction time, confidence, and the chosen response**

Here we repeat the analyses looking at the effects of response, validity, and believability on reaction time and confidence. The posterior estimates, HDI, and proportions of credible values are summarised in Table 5 (analogous to Table 5 in main text that contains frequentist results). Both reaction time and confidence models showed similar results to frequentist analyses – there is good evidence to support the effect of response and the interaction between response and believability on reaction time, and good evidence to support the effect of response, believability, and the interaction between believability and response on confidence.

*Table 5: Predicting log-RT and confidence based on response (yes, no), item validity (invalid, valid), and item believability (unbelievable, believable), showing Bayesian analyses. OR represents the estimate, HDI the 95% credibility interval, and CV represents the proportion of credible values within the HDI that fall in the same direction as the estimate.*

|  | Log-RT | | | Confidence | | |
| --- | --- | --- | --- | --- | --- | --- |
| Predictors | OR | HDI | CV | b | HDI | CV |
| Response (No) | **1.10** | **1.06, 1.16** | **0.99** | **-4.05** | **-5.50, -2.62** | **1.0** |
| Validity (Invalid) | 1.04 | 0.97, 1.13 | 0.25 | -0.79 | -1.73, 0.10 | 0.96 |
| Believability (Unbelievable) | 0.99 | 0.92, 1.07 | 0.82 | **-1.56** | **-2.67, -0.49** | **0.99** |
| Response * Validity | 0.99 | 0.96, 1.03 | 0.98 | 0.46 | -0.53, 1.47 | 0.82 |
| Response * Believability | **0.92** | **0.88, 0.95** | **0.99** | **3.41** | **2.24, 4.58** | **1.0** |
| Validity * Believability | 1.00 | 0.93, 1.08 | 0.16 | 0.09 | -0.80, 0.99 | 0.57 |
| Response * Validity * Believability | 1.00 | 0.96, 1.04 | 0.37 | -0.30 | -1.25, 0.64 | 0.7 |

**Experiment 2**

**(3.2.1) What is the intuitive response to AC and DA conditional syllogisms?**

First, the analyses suggest there is not a credible difference between responses on valid and invalid items in the speeded abstract task (*OR* = 0.95, *HDI* = 0.75, 1.20; *CV* = 0.67).

Second, we modelled responses on the main task as predicted by proportion of conclusion endorsements on the speeded abstract task and item validity. Participants were more likely to endorse the conclusions on the main task items when they endorsed more conclusions on the speeded abstract task (*OR* = 11.77, *HDI* = 7.65, 18.11, *CV* = 1.00), were less likely to endorse conclusions on invalid than valid items (*OR* = 0.38, *HDI* = 0.28, 0.53, *CV* = 1.00), and there was insufficient evidence for an interaction effect (*OR* = 1.18, *HDI* = 0.90, 1.53, *CV* = 0.89). Pairwise contrasts further showed that participants were more likely to endorse the conclusion of main task items as valid when they endorsed more of the abstract items as valid, among both valid (*OR* = 9.97, *HDI* = 5.36, 15.7) and invalid items (*OR* = 13.81, *HDI* = 7.94, 21.8). These results replicate the frequentist analyses.

**(3.2.2) Relationship between reaction time, confidence, and the chosen response**

We repeated the models looking at the effects of response (yes, no), validity, and believability on reaction time and confidence with a Bayesian approach. The effects are summarised in Table 9. In the reaction time model, there were credible interactions between response and validity (*OR* = 1.06, *HDI* = 1.02, 1.09; *CV* = 0.99), and between response and believability (*OR* = 0.92, *HDI* = 0.89, 0.96; *CV* = 1.0). In the confidence model, there was a credible effect of response, so that ‘no’ responses were given with less confidence than ‘yes’ responses (*b* = -3.98, *HDI* = -5.07, -2.92; *CV* = 1.0), a small interaction effect between response and validity (*b* = 1.00, *HDI* = 0.18, 1.85; *CV* = 0.99), and a larger interaction between response and believability (*b* = 3.96, *HDI* = 2.92, 5.00; *CV* = 1.0). Other posterior estimates included many non-credible values, so we consider them to be absent.

The results were very similar to frequentist analyses both with reaction time and confidence. Valid and invalid items were generally answered with the same speed and confidence – with the exception of frequentist analyses of reaction time, but not the Bayesian analyses, which detected no main effect of validity.

*Table 9: Predicting log-RT and confidence based on response (yes, no), item validity (invalid, valid), and item believability (unbelievable, believable). Includes both standard and neutral items.*

|  | Log-RT | | | Confidence | | |
| --- | --- | --- | --- | --- | --- | --- |
| Predictors | OR | HDI | CV | b | HDI | CV |
| Response (No) | 1.03 | 1.00, 1.07 | 0.96 | **-3.98** | **-5.07, -2.92** | **1.0** |
| Validity (Invalid) | 1.04 | 0.98, 1.09 | 0.91 | -0.13 | -0.85, 0.60 | 0.64 |
| Believability (Unbelievable) | 1.05 | 0.99, 1.10 | 0.96 | -0.19 | -1.00, 0.64 | 0.67 |
| Response * Validity | **1.06** | **1.02, 1.09** | **0.99** | **1.00** | **0.18, 1.85** | **0.99** |
| Response * Believability | **0.92** | **0.89, 0.96** | **1.0** | **3.96** | **2.92, 5.00** | **1.0** |
| Validity * Believability | 0.99 | 0.94, 1.04 | 0.67 | 0.09 | -0.56, 0.76 | 0.61 |
| Response * Validity * Believability | 1.00 | 0.97, 1.03 | 0.62 | 0.35 | -0.46, 1.16 | 0.81 |
